# CCL5 promotes angiotensin II-induced cardiac remodeling through regulation of platelet-driven M2 macrophage polarization

**DOI:** 10.7150/thno.112163

**Published:** 2026-01-01

**Authors:** Silin Lv, Mingxuan Zhou, Tiegang Li, Zifan Zeng, Zheng Yan, Yufang Hou, Liu Yang, Fang Zhang, Wenyi Zhao, Yixin Zhou, Min Yang

**Affiliations:** 1 State Key Laboratory of Bioactive Substance and Function of Natural Medicines, Institute of Materia Medica, Chinese Academy of Medical Sciences and Peking Union Medical College, Beijing 100050, China.; 2 State Key Laboratory of Digestive Health, Institute of Materia Medica, Chinese Academy of Medical Sciences and Peking Union Medical College, Beijing 100050, China.

**Keywords:** CCL5, hypertension, cardiac remodeling, platelet inflammation, M2 macrophage

## Abstract

**Rationale:** Sustained hypertension induces adverse cardiac remodeling. Platelet activation is instrumental in exacerbating inflammation by engaging with macrophages. C-C chemokine motif ligand 5 (CCL5) is contained within platelet α-granules, and released following platelet activation. This work delineated the specific contributions of CCL5 to platelet function, platelet-induced macrophage polarization, and hypertensive cardiac remodeling.

**Methods:** CCL5 knockout (KO) mice infused with Angiotensin II (Ang II) were used to identify the role of CCL5 *in vivo*. CCL5 absence on platelet activation were evaluated on washed platelets. Two distinct models of platelet depletion and reconstitution were utilized to investigate the impact of platelets lacking CCL5. An *in vitro* co-culture system was established to explore the roles of CCL5-mediated platelet activation in M2 macrophage polarization.

**Results:** CCL5 KO attenuated the adverse cardiac effects induced by Ang II, including fibrosis, hypertrophy, and functional impairment, accompanied by reduced platelet activation and M2 macrophage polarization in cardiac tissue. Platelet inhibitor administration and platelet depletion/reconstitution experiments revealed that the suppression of platelet activation by CCL5 KO contributed to the amelioration of Ang II-promoted cardiac M2 macrophage polarization and cardiac remodeling. CCL5 KO markedly suppressed the activation of TGF-β1 and NF-κB signaling, an effect observed both in cardiac tissue from Ang II-infused mice and in platelets following ADP stimulation *in vitro*. In *in vitro* co-culture systems, rmTGF-β1 reversed CCL5 KO platelet-impaired M2 macrophage polarization. NF-κB inhibition abolished recombinant CCL5 (rmCCL5)-induced platelet activation, while blocking antibodies against CCR1 and CCR3 inhibited rmCCL5-induced NF-κB signaling and platelet activation.

**Conclusions:** These findings underscore the detrimental role of CCL5-mediated platelet activation in promoting M2 macrophage polarization during hypertensive cardiac remodeling and elucidate the molecular mechanism that CCL5 facilitates platelet-derived TGF-β1 signaling by promoting NF-κB activation *via* CCR1 and CCR3 receptors. These findings support CCL5 inhibition as a promising strategy against inflammation and cardiac damage.

## Introduction

Hypertension-induced adverse cardiac remodeling, characterized by left ventricular (LV) hypertrophy and fibrosis, is a major driver of progression toward heart failure 1. Angiotensin II (Ang II) contributes significantly to the development of hypertension and drives the process of cardiac remodeling 2. Ang II triggers a series of cardiac inflammatory responses, including immune cell recruitment, pro-fibrotic M2 macrophage polarization, and upregulation of inflammatory cytokines 3-5. Research indicates that inhibiting key inflammatory mediators or modulating immunocytes can mitigate cardiac remodeling prompted by Ang II 4, 5. Therefore, a deeper understanding of the underlying immuno-inflammatory pathways in hypertensive cardiac remodeling is essential for preventing and treating hypertensive cardiac injury and heart failure.

Accumulating evidence points to the essential involvement of platelets in inflammatory processes, including their interaction with, stimulation of, and regulation of T cells, neutrophils, monocytes, and macrophages 6. Platelets contribute to hypertensive organ damage by modulating the inflammatory response 7. In response to circulatory changes such as endothelial cell damage, vessel wall injury, or hemodynamic abnormalities, platelets undergo morphological changes and modulate inflammation by secreting factors and interactions with inflammatory cells 6. Using experimental models of pressure overload, ventricular fibrosis is associated with platelet-derived TGF-β1 8. Pharmacological inhibition of platelets or genetic TGF-β1 depletion from platelets markedly alleviates structural alterations in the atrium induced by Ang II with reduced inflammatory damage in the atrium in hypertensive models 9. Depletion of platelets *via* anti-GP1bα antibody, blockade of platelet P-selectin, or inhibiting platelet with clopidogrel effectively limits the interaction between activated platelets and leukocytes, preventing cardiac remodeling in transverse aortic constriction (TAC)-induced hypertensive models 7. Furthermore, platelet activation has been shown as an early event during hypertension induced by Ang II, stimulating the conjugation between platelets and leukocytes and promoting the recruitment of inflammatory cells into cardiac tissue, where they contribute to cardiac fibrosis by secreting inflammatory cytokines 10. Consistent with this, several studies have emphasized platelets as a source of cytokines that may drive macrophage polarization toward a fibrogenic phenotype, thereby activating fibroblasts and promoting cardiac remodeling 11. Nevertheless, how platelets contribute to Ang II-induced cardiac remodeling remains poorly defined, and the effector molecules triggering platelet activation in this context are not well characterized. Additionally, the potential cooperation between platelets and macrophages during the development of cardiac remodeling warrants further investigation.

C-C chemokine motif ligand 5 (CCL5), also known as RANTES, is a chemokine that regulates monocytes and T cells migration during various pathological processes 12, 13. CCL5 is abundant in platelet α-granules, and is secreted upon platelet activation 14. Both CCL5 receptors, CCR1 and CCR3, are expressed on platelets 15. However, the role of CCL5 on platelet function remains poorly understood. Substantial evidence suggests that CCL5 is implicated in inflammatory diseases, including viral infections, asthma, atherosclerosis, and liver fibrosis 16. Pharmacological or genetic interventions targeting CCL5 attenuate the infiltration of T lymphocytes and macrophages into perivascular adipose tissue induced by Ang II, thereby enhancing vascular performance 17. In contrast, CCL5 knockout (KO) mice exhibit exacerbated kidney damage compared to wild-type (WT) controls with Ang II infusion, which results from increased macrophage infiltration, and heightened pro-inflammatory cytokine production 18. These observations reveal that CCL5 plays a complex and pivotal role in end-organ damage in hypertension. Given the central involvement of platelets in cardiovascular pathology and the significance of autocrine mechanisms in controlling platelet function, this study investigated whether CCL5 triggers platelet activation to influence hypertensive cardiac remodeling.

The present work clarifies the contribution of CCL5 to inflammatory responses mediated by platelets during cardiac remodeling induced by Ang II. The results elucidate a previously unrecognized pathway through which CCL5 activates platelets through CCR1 and CCR3 receptors to promote activation of nuclear factor-kappa B (NF-κB) signaling. This activation, subsequently, regulates the secretion of TGF-β1 derived from platelets, ultimately promoting the differentiation of macrophages towards the M2 phenotype. Additionally, CCL5 deficiency prevents platelet activation, platelet-driven M2 macrophage polarization, and cardiac remodeling in Ang II-infused mice. This work offers new perspectives on platelet biology, emphasizing how CCL5 enhances platelet activation and promotes a pro-fibrotic macrophage phenotype during hypertension-induced cardiac remodeling.

## Materials and Methods

### Experimental animals

The congenic CCL5 KO mice, male and 10-12 weeks old, were derived from the C57BL/6J strain using CRISPR/Cas9 technology targeting exons 1 to 3 of the CCL5 gene. A 5214 bp deletion, accompanied by a point mutation (G>A), was introduced in this region, resulting in a premature termination of translation. The positive founder was crossed into C57BL/6J mice to generate heterozygous individuals, which were subsequently intercrossed to produce the CCL5 KO mice. Genotypes (Figure S1A) were confirmed *via* DNA sequencing of tail samples using the forward sequencing primer: GATAATAGATGGACATAGAGGACACAACTC (Figure S1B). All animal protocols were reviewed and approved by the Animal Care and Use Committee at the Institute of Materia Medica, Chinese Academy of Medical Sciences & Peking Union Medical College, ensuring full adherence to the ARRIVE guidelines. (Approval no. 00003901).

### Hypertensive mice model established* via* Ang II infusion

To induce hypertension, mice received a continuous infusion of angiotensin II (Ang II, Sigma-Aldrich, St. Louis, MO, USA) at a rate of 1500 ng·kg⁻¹·min⁻¹ for 7 days 19. Following assessment of hemodynamic parameters, mice were anesthetized with 2% isoflurane and euthanized. Isolated hearts were excised and processed for histological, RNA sequencing, and molecular analyses. All animals were assigned an alphanumeric designation for blinding purposes.

### Aspirin (ASA) and clopidogrel (CPG) treatment

ASA (Sigma-Aldrich) and CPG (MedChemExpress, NJ, USA) were dissolved separately in 300 mL of drinking water (0.4 mg/mL ASA, 0.15 mg/mL CPG) and provided with free access for 24 hours 20. The drinking water was replaced daily. Pre-treatment with ASA or CPG was initiated three days prior to the start of Ang II administration and maintained during the entire period of Ang II challenge.

### Platelet depletion/reconstitution model

Busulfan model: Busulfan (HY-B0245, MedChemExpress), a selective bone marrow toxin, was used to induce platelet depletion 21. On days 0 and 3, mice were administered busulfan (20 mg/kg) or vehicle intraperitoneally. After 14 days, washed platelets were injected intravenously into the platelet-depleted mice 20 minutes prior to Ang II infusion. The injection was repeated on day 3 post-surgery. The injection volume of 200 μL contained 5 × 10^8^ platelets. Busulfan administration did not significantly change the quantity of peripheral blood leukocytes, which was verified using flow cytometric analysis (Figure S2).

Antibody model: One intraperitoneal injection of a GPIbα-blocking antibody (0.3 μg/g, R300; Emfret Analytics, Bayern, Germany) was administered to the mice. After 24 hours, washed platelets were injected intravenously into the platelet-depleted mice 20 minutes prior to Ang II infusion. The injection was repeated on day 3 post-surgery. The injection volume of 200 μL contained 1 × 10^9^ platelets.

### Immunohistochemistry and immunofluorescence

Immunohistochemistry of cardiac sections was conducted with primary antibodies against α-SMA (1:200; ab5694, Abcam Cambridge, MA, USA), MAC-2 (1:200; ab76245, Abcam Cambridge), CD8 (1:1000; ab217344, Abcam Cambridge), CD163 (1:100; ab182422, Abcam Cambridge), and CD41 (1:50; sc-365938, Santa Cruz Biotechnology, CA, USA), ARG-1 (1:200; 16001-1-AP, Proteintech, Wuhan, China). A semiquantitative assessment of staining was performed, incorporating both intensity (0: none; 1: mild; 2: moderate; 3: intense) and the percentage of positive cells (0: 0%; 1: 1-25%; 2: 26-50%; 3: 51-75%; 4: 76-100%). Immunofluorescence of cardiac sections was conducted with primary antibodies against CCL5 (1:100; 710001, Sigma-Aldrich), CD41 (1:50; sc-365938, Santa Cruz Biotechnology), α-SMA (1:200; ab5694, Abcam Cambridge), iNOS (1:100; 18985-1-AP, Proteintech), ARG-1 (1:200; 16001-1-AP, Proteintech), CD68 (1:100; 38005, Signalway Antibody, Beijing, China), MAC-2 (1:200; ab76245, Abcam Cambridge), CD8 (1:500; ab217344, Abcam Cambridge), α-actin (1:200; A5044, Sigma-Aldrich), CD31 (1:200; 77699S, Cell Signaling Technology), and TGF-β1 (1:50, sc-130348, Santa Cruz Biotechnology; 1:100, ab92486, Abcam Cambridge). Cardiomyocyte cross-sectional area was detected by wheat germ agglutinin (WGA; Invitrogen, Waltham, MA, USA). Immunofluorescence of macrophages and platelets was performed with primary antibodies targeting CCR1 (1:100; 54526, Signalway Antibody), CCR2 (1:100; bs-0562R, Bioss, Beijing, China) and CCR3 (1:100; 56092, Signalway Antibody). Analysis was conducted with a confocal laser scanning microscope (Leica, Wetzlar, Germany).

### Cardiac magnetic resonance imaging (CMRI)

CMRI studies were performed on mouse hearts using a 7.0 T Bruker BioSpec system (Bruker Medical, Ettlingen, Germany), which was fitted with a small animal coil. Short-axis cine images of the left ventricle were obtained. Throughout the cardiac cycle, fifteen phases were captured, acquiring continuous slices from the cardiac base to the apex. ParaVision 6.0.1 (Bruker BioSpec, Bruker Medical) was used for image analysis.

### Blood pressure measurement

Arterial blood pressure was measured using a non-invasive tail-cuff system (Softron BP-98A, Tokyo, Japan). Experiments were conducted in a quiet, darkened room (22 ± 2°C), with access restricted to the operator during measurements. To minimize stress, mice were acclimated to the restraint tubes, and trained to voluntarily enter the restraint tubes during measurement. Mice were subjected to a standardized warming protocol of 35°C for 5 minutes prior to data acquisition and were maintained at this temperature throughout the measurement period. Following an initial warm-up period of 10 cycles, data from the subsequent 5 to 15 cycles (of a total 15-25) per session were retained for analysis, and these cycles were used to calculate average blood pressure for statistical comparisons. To ensure measurement accuracy and reliability, mice were acclimated to the tail-cuff procedure through a training period of at least five consecutive days prior to experimental data collection.

### RNA sequencing analysis

RNA sequencing of ventricular tissue was conducted by an Illumina HiSeq4000 system (Expandbio, Beijing, China). PCA analysis was conducted using the scatterplot3d in R to reveal the interrelationship between the samples. Differentially expressed genes (DEGs) were discerned between saline-treated WT and Ang II-infused WT hearts, as well as between Ang II-treated WT and Ang II-infused CCL5 KO hearts, *via* the 'limma' package in R, considering an adjusted P-value < 0.05 as statistically significant. The R-based tool "clusterProfiler" was employed to conduct KEGG, GO, along with GSEA, regarding an FDR-adjusted P-value below 0.05 as significant. Immune infiltration was evaluated with five different methods: murine Microenvironment Cell Population counter (mMCP-counter) 22, Estimating the Proportion of Immune and Cancer Cells (EPIC) 23, Tumor Immune Estimation Resource (TIMER) 24, 25, Tumor Immune contexture from human RNA-seq data (quanTIseq) 26, and Celltype Identification By Estimating Relative Subsets of RNA Transcripts (CIBERSORT-ABS) 27.

### GEO dataset analysis

RNA-seq datasets for both control and Ang II-infused groups (16 samples per group) were acquired from the Gene Expression Omnibus (GEO) repository (GSE261273 and GSE261275). DEGs were determined using a corrected P-value threshold of 0.05. Correlations in transcript levels were assessed using Spearman's method, considering a P-value below 0.05 and an absolute correlation coefficient exceeding 0.3 as statistically significant.

### Molecular docking analysis

To explore the binding between CCL5 and its receptors CCR1 and CCR3, rigid protein-protein docking was carried out with ZDOCK. The three-dimensional structures of CCL5, CCR1, and CCR3 were acquired from the Protein Data Bank (PDB). Protein-protein interactions were examined and visualized using PyMol (Version 2.4).

### Platelet isolation

Cardiac puncture was performed to collect whole blood into acid-citrate-dextrose (1:7). Platelet rich plasma was obtained *via* centrifugation for 11 minutes at 200 g and room temperature. After washing with two rounds of CGS solution containing PGE1 (1 μM; HY-B0131, MedChemExpress), platelets were finally suspended in a modified Tyrode's solution to a density of 3 × 10^8^/mL. Platelets were allowed to recover to a resting state by incubating at room temperature for 1 hour.

### Spreading of platelets

Fibrinogen (10 μg/mL; HY-125864, Sigma-Aldrich) was applied to cell culture plates with glass coverslips overnight at 4°C. The platelets were left for two hours at 37°C to allow adhesion and spreading on the fibrinogen-coated surfaces. After fixation and permeabilization, attached platelets were stained with FITC-labeled phalloidin (CA1620, Solarbio, Beijing, China). Confocal laser scanning microscopy (Leica) was used to capture images, and ImageJ software was used to calculate each platelet's spreading area.

### Flow cytometry

Washed platelets were incubated with FITC anti-mouse CD41 antibody (133904; Biolegend, San Diego, CA, USA) and APC anti-mouse/rat CD62P (148304; Biolegend) for 15 minutes. In inhibitor experiments, washed platelets were pre-treated with or without aspirin (2 mM; A2093, Sigma-Aldrich), BAY 11-7082 (1 μM; HY-13453, MedChemExpress), CCR1 blocking antibody (50 μg/mL, K115510P-Ag, Solarbio, Beijing, China) or CCR3 blocking antibody (50 μg/mL, K113920P-Ag, Solarbio, Beijing, China) for 15 minutes before stimulation with or without rmCCL5 (100 ng/mL; 250-07, PeproTech) in the presence of ADP (5 μM; A2754, Sigma-Aldrich) for 10 minutes. The specificity of blocking antibodies was confirmed using both bone marrow-derived macrophages (BMDMs) and platelets (Figure S3). BMDMs treated with CCR1 and CCR3 blocking antibodies were stained with APC anti-mouse CCR1 (152503), PE/Cyanine7 anti-mouse CCR2 (150611) and APC anti-mouse CCR3 (144511) (all from Biolegend) for 30 minutes at 4 °C.

For peripheral blood leukocyte subsets quantification, 100 μL of peripheral blood was stained with the following antibodies: PE/Dazzle™ 594 anti-CD45 (103146), PE anti-CD11b (101208), APC anti-F4/80 (157305), FITC anti-Ly-6G (127605), PE anti-CD3 (155608), APC anti-CD4 (100516), FITC anti-CD8a (100706), and APC anti-CD19 (115512) (all from BioLegend). Then erythrocytes were lysed by incubating the samples with 1 mL of BD FACS lysing solution (349202; BD Biosciences, San Jose, CA, USA) per 100 μL of blood.

To identify cardiac macrophage subpopulations, mice were euthanized and hearts were perfused with heparinized saline (2% heparin) to clear residual blood. Cardiac tissues were dissociated enzymatically using a mixture of dispase II (2.4 U/mL) and collagenase type II (0.01 mg/mL) for 60 minutes at 37 °C. The resulting single-cell suspensions were sequentially filtered through 70-μm and 40-μm strainers. To minimize nonspecific antibody binding, cells were pre-blocked with an anti-CD16/32 antibody and subsequently stained with a Fixable Viability Dye and fluorescently labeled antibodies: PE/Dazzle™ 594 anti-mouse CD45 (103146), APC anti-mouse F4/80 (157305), PE anti-mouse CD86 (105008), and PE/Cyanine7 anti-mouse CCR2 (150611) (all from BioLegend). Following washing and centrifugation, cells were fixed, permeabilized, and stained with FITC anti-mouse CD206 (141703; BioLegend).

ImageStream Mark II imaging flow cytometry (Amnis, EMD-Millipore, Seattle, WA, USA) was used for sample analysis, and IDEAS statistical image analysis software (Amnis, EMD-Millipore, Seattle, WA, USA) was used for data processing.

### Bone marrow-derived macrophages (BMDMs)

Bone marrow cells were flushed from femurs and tibias with DMEM using a 1 mL syringe and then isolated *via* Ficoll-Paque density gradient centrifugation. Macrophages were polarized from bone marrow cells treated with 10 ng/mL M-CSF (PeproTech) for 3 days, and proliferated in new media for another 2 days before use for experiments 3. BMDMs were co-cultured with wild-type (WT) or CCL5 knockout (KO) mouse-derived platelets at a ratio of 1:1000, along with ADP (10 μM) and Ang II (100 nM) for 48 hours. Polarization toward the M2 phenotype was evaluated using immunofluorescence and real-time PCR. Culture supernatants were collected for subsequent fibroblast stimulation experiments.

### Generation of M1 and M2 macrophages

RAW264.7 cells were polarized into M1 macrophages through stimulation with lipopolysaccharide (LPS, 100 ng/mL; L2880, Sigma-Aldrich) and interferon-gamma (IFN-γ, 20 ng/mL; AF-315-05, PeproTech). For M2 polarization, cells were treated with IL-4 (20 ng/mL; AF-214-14, PeproTech) and IL-13 (20 ng/mL; 210-13, PeproTech), while control groups (M0) received only culture medium. All treatments were carried out for 48 hours *in vitro*.

### Cardiac fibroblasts

In brief, heart tissues from neonatal Sprague-Dawley rats were minced and subjected to sequential digestions in a solution of 0.1% trypsin and 100 U/mL collagenase type II at 37 °C. Filtration was performed on the supernatant using a 70-μm cell strainer. The final resuspended cells were plated for 90 minutes to facilitate fibroblast adhesion. Before subsequent experiments, cells were cultured in DMEM with 5% FBS for 24 hours. To induce myofibroblast differentiation, cells were stimulated by conditioned media from M1 macrophages, M2 macrophages, or platelet-educated BMDMs for 48 hours. Differentiation was assessed *via* immunofluorescence and RT-PCR.

### Enzyme-linked immunosorbent assay (ELISA)

TGF-β1 levels were detected using a Mouse TGF-β1 pre-coated ELISA kit (1217102; DARKEWE, Shenzhen, China) or a Rat TGF-β1 ELISA kit (ERC107b.48; Neobioscience Technology Co, Ltd., Shenzhen, China).

### Western blotting analysis

SDS-PAGE of 10%, 15%, or 20% was used for protein separation. Primary antibodies against CCL5 (1:1000; 710001, Sigma-Aldrich), MRC-1 (1:1000; ab646963, Abcam, Cambridge, MA, USA), TGF-β1 (1:500; sc-130348, Santa Cruz Biotechnology), P65 (1:1000; 8242T, Cell Signaling Technology, Danvers, MA, USA), p-P65 (1:1000; 11014, Signalway Antibody, Beijing, China), and GAPDH (1:1000; 5174s, Cell Signaling Technology) were used. Protein signals were visualized by enhanced chemiluminescence (ECL; P90720, EMD-Millipore, Seattle, WA, USA) following incubation with species-matched secondary antibodies, and images were acquired using an ImageQuant™ LAS 4000 system (GE, Boston, USA).

### Real-time PCR (RT-PCR)

The commercial RNA Tissue/Cell Rapid Extraction Kit (Shandong Sparkjade Biotechnology Co., Ltd., China) was used for total RNA extraction. Reverse transcription was conducted with SPARKscript ll RT Plus Kit (Shandong Sparkjade Biotechnology Co., Ltd.). Quantitative PCR was performed using SPARKscript Ⅱ SYBR One Step qRT-PCR Kit (Shandong Sparkjade Biotechnology Co., Ltd.) and specific primers on an Applied Biosystems 7900 PCR system (ABI, Carlsbad, CA, USA) (Table S1). 2^-ΔΔCt^ method was used for RNA expression analysis with GAPDH as the endogenous reference gene.

### Statistical analysis

Data were presented as mean ± SD. The distribution of data was assessed by Shapiro-Wilk normality test. For normally distributed data, Student's t-test was applied for two-group comparisons, and one-way ANOVA followed by Tukey's post-hoc test was used for comparisons between three or more groups. For non-normally distributed variables, Mann-Whitney test was applied for comparisons between two groups, while the Kruskal-Wallis test with Dunn's post hoc analysis was applied for comparisons among multiple groups. (*p < 0.05, **p < 0.01, ***p < 0.001, NS indicating no significant difference).

## Results

### CCL5 deficiency attenuated Ang II-promoted cardiac fibrosis, hypertrophy, and dysfunction

**Figure 1:**
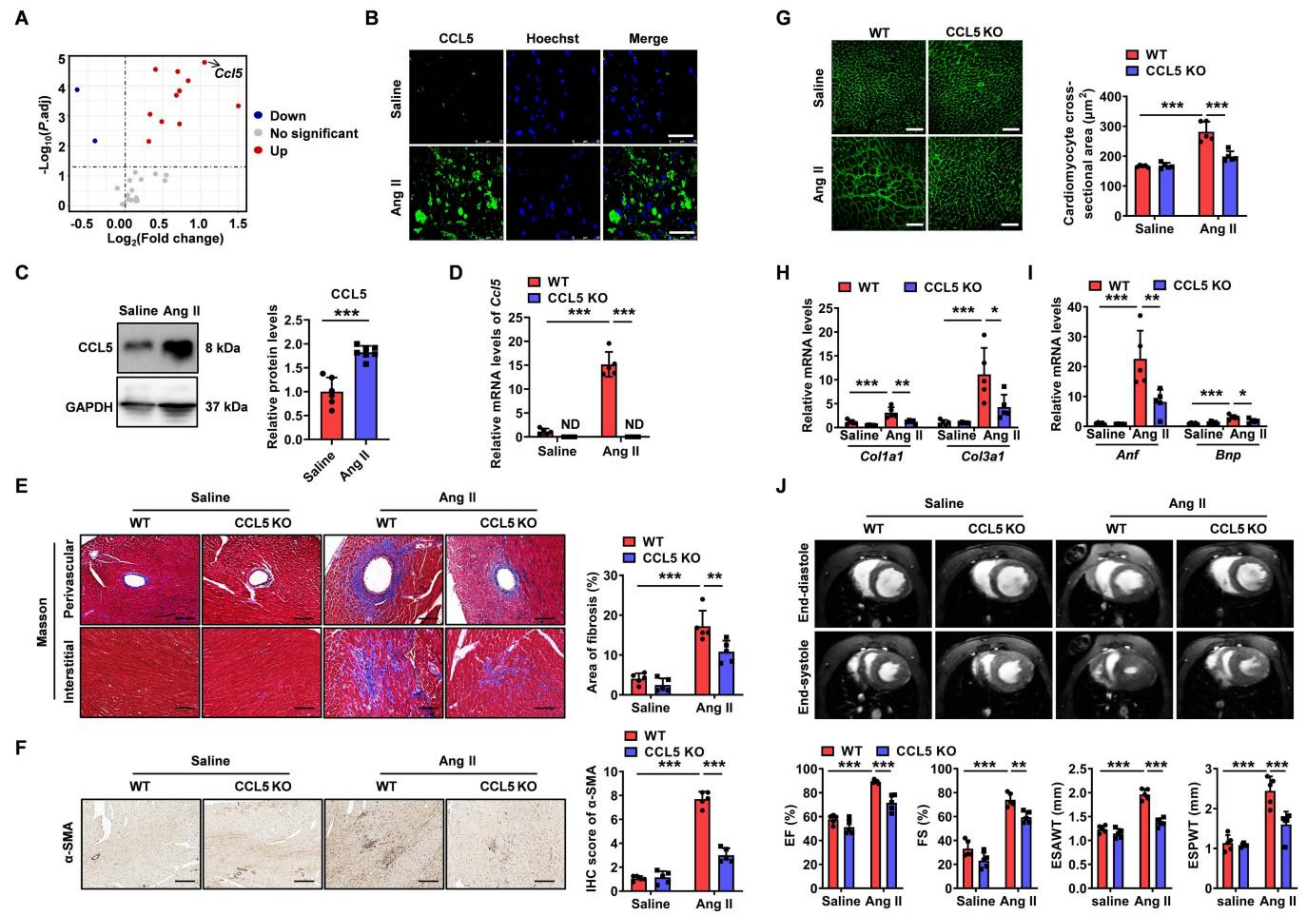
** CCL5 deficiency attenuated Ang II-promoted cardiac fibrosis, hypertrophy, and dysfunction.** Fold change and statistical analysis of chemokine transcript levels in hearts exposed to angiotensin II (Ang II), compared with saline-treated controls at day 7, based on GEO datasets (n = 16 per group). Wild-type (WT) mice received a 7-day infusion of either saline or Ang II. **B** CCL5 (green) in the heart detected by immunofluorescence (n = 5; Scale bar: 25 μm). **C** CCL5 protein levels in the hearts detected by western blotting analysis of (n = 6 for saline and n = 7 for Ang II groups). WT and CCL5 KO mice were subjected to a 7-day infusion of saline or Ang II (n = 5 per group). **D** The gene expression of *Ccl5* in heart tissues assessed by RT-PCR. **E** Myocardial fibrosis detected by Masson's trichrome staining (Scale bar: 100 μm). **F** Immunohistochemistry of α-SMA in the heart (Scale bar: 100 μm). **G** FITC-WGA staining showed cardiomyocyte cross-sectional area (Scale bar: 50 μm). **H, I** Genes expression of* Col1a1*, *Col3a1*, *Anf*, and *Bnp* in heart tissues. **J** CMRI analysis of left ventricle at end-systole and end-diastole. Data are presented as mean ± standard deviation, with n representing the number of animals. * P < 0.05, ** P < 0.01, *** P < 0.001, ND indicating no detection and NS indicating no significance.

To evaluate the contribution of CCL5 to Ang II-induced cardiac remodeling, chemokine gene expression was assessed in cardiac tissue from Ang II-infused mice at day 7. Two mRNA sequencing datasets (GSE261273 and GSE261275) were retrieved from the GEO database, comprising a total of 32 samples—16 from saline-treated controls and 16 from Ang II-infused animals 28. Of the 29 chemokine genes examined, *Ccl5* showed the most marked induction in cardiac tissue following Ang II infusion when compared to sham-treated animals (Figure 1A), highlighting its central role in cardiac remodeling processes driven by Ang II. Pathway enrichment and gene set enrichment analysis (GSEA) based on GEO datasets revealed multiple signaling pathways regulating CCL5 expression were activated in Ang II-infused heart (Figure S4A). These pathways included the mitogen-activated protein kinases (MAPK), Janus kinase/signal transducer and activator of transcription (JAK-STAT), nuclear factor-kappa B (NF-κB), and the phosphatidylinositol 3-kinase (PI3K)/Akt signaling pathway 29-31. A strong positive correlation between the scores of these pathways and *Ccl5* gene expression was demonstrated by gene set variation analysis (GSVA) (Figure S4B). The upregulation of CCL5 protein in Ang II-infused hearts was subsequently confirmed through both immunofluorescence and western blotting analysis (Figure 1B, C).

To evaluate the functional role of CCL5 in Ang II-induced hypertensive cardiac remodeling, wild-type (WT) and CCL5 knockout (CCL5 KO) mice were examined. Quantitative PCR measurements verified elevated cardiac *Ccl5* transcript levels in WT mice following Ang II infusion, while confirming its absence in CCL5 KO animals (Figure 1D). Genetic deletion of CCL5 did not alter hypertension triggered by Ang II (Figure S5). Histological evaluation with Masson's trichrome revealed that CCL5 KO markedly reduced both perivascular and interstitial fibrosis induced by Ang II, along with a decrease in α-smooth muscle actin (α-SMA)-positive myofibroblast accumulation (Figure 1E, F). Cardiac hypertrophy promoted by Ang II, assessed *via* cardiomyocyte cross-sectional area, was also significantly suppressed in CCL5 KO mice (Figure 1G). Furthermore, mRNA expression of fibrotic and hypertrophic markers—collagen I (*Col1a1*), collagen III (*Col3a1*), atrial natriuretic factor (*Anf*), and brain natriuretic peptide (*Bnp*)—was notably lower in CCL5 KO compared to WT mice after Ang II challenge (Figure 1H, I). Cardiac MRI assessments indicated that CCL5 deficiency alleviated Ang II-impaired contractility, reflected by recovered left ventricular ejection fraction (EF%) and fractional shortening (FS%), as well as reduced end-systolic anterior and posterior wall thicknesses relative to WT mice (Figure 1J). Taken together, these data indicate a critical function for CCL5 in driving Ang II-induced cardiac fibrosis, hypertrophy, and ventricular dysfunction.

### Transcriptomic profiling reveals CCL5-mediated regulation in hypertensive cardiac remodeling

**Figure 2:**
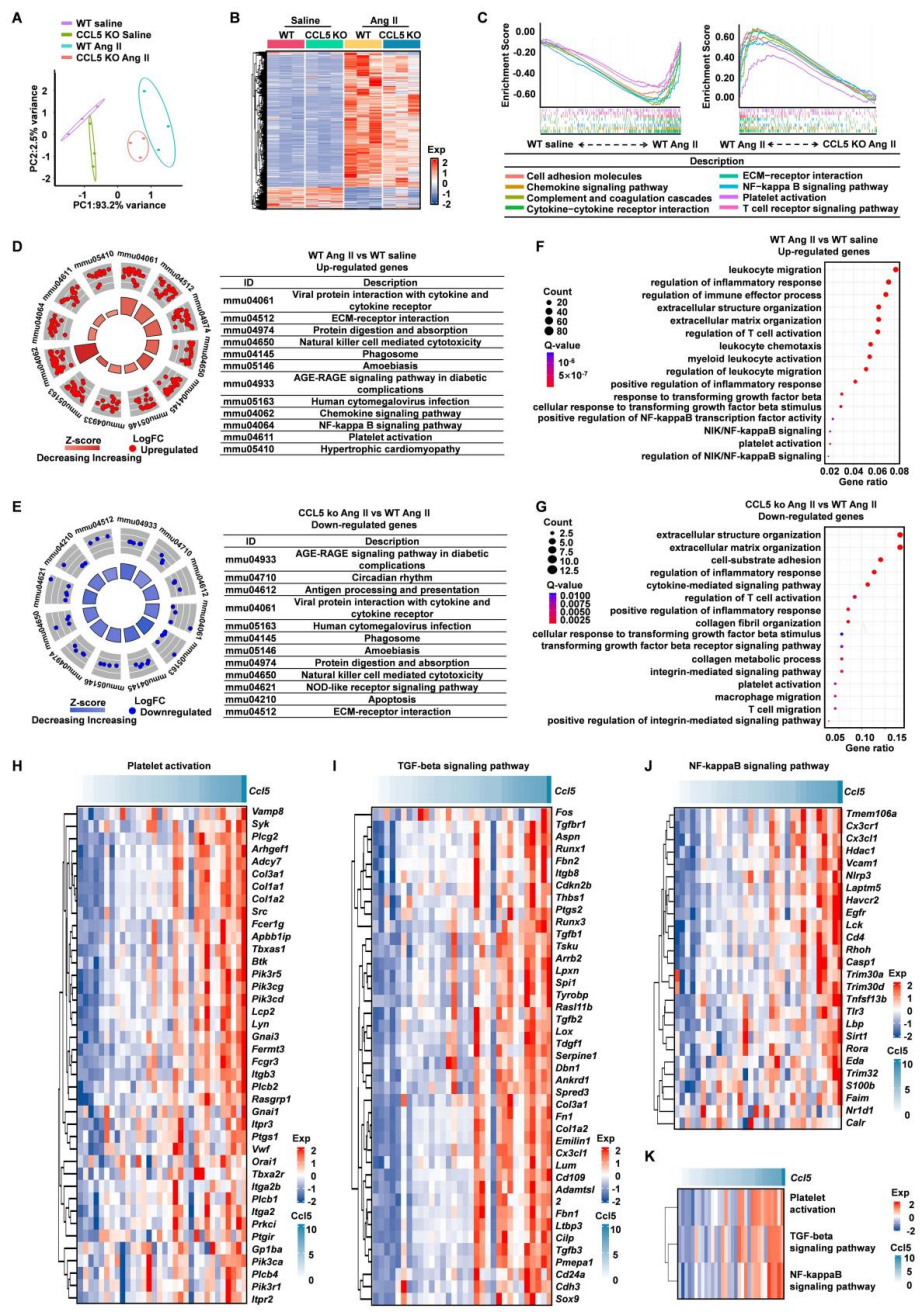
** Transcriptomic profiling reveals CCL5-mediated regulation in hypertensive cardiac remodeling.** Cardiac tissues from WT and CCL5 KO mice infused for 7 days with saline or Ang II were analyzed *via* RNA sequencing (n = 3 per group). **A** Principal component analysis (PCA) illustrating distinct transcriptomic profiles among the four experimental groups. **B** Heatmap of differentially expressed genes (DEGs) identified in WT Ang II vs. WT saline hearts, and in CCL5 KO Ang II vs. WT Ang II hearts. **C** Gene Set Enrichment Analysis (GSEA) highlighting biological pathways enriched in WT Ang II hearts relative to both saline-treated WT and CCL5 KO Ang II groups. **D, E** KEGG pathway analysis of up-regulated genes under Ang II stimulation in WT mice (**D**) and down-regulated genes in CCL5 KO Ang II vs. WT Ang II hearts (**E**). **F, G** Significantly enriched Gene Ontology (GO) terms for up-regulated genes in WT Ang II vs. saline controls (**F**) and down-regulated genes in CCL5 KO Ang II vs. WT Ang II hearts (**G**). **H-J** Correlation analysis in WT mice between *Ccl5* expression and key genes involved in platelet activation, TGF-β, and NF-κB signaling under Ang II or saline conditions (GEO datasets; n = 32). **K** Correlation of *Ccl5* transcript levels with Gene Set Variation Analysis (GSVA) scores for platelet activation, TGF-β, and NF-κB pathways following Ang II or saline infusion (n = 32).

To uncover the transcriptional mechanisms through which CCL5 deficiency confers protection against hypertensive cardiac remodeling, we carried out RNA-seq on heart tissues from WT and CCL5 KO mice following infusion with saline or Ang II. Principal component analysis (PCA) illustrated clear separation among all groups, with high reproducibility within replicates and condition-specific transcriptomic signatures (Figure 2A). Ang II challenge led to substantial transcriptional activation in WT hearts, which was markedly reduced in CCL5 KO mice, implying that CCL5 loss restrains Ang II-induced gene expression changes (Figure 2B). GSEA was employed to identify processes modulated by CCL5. In WT animals, Ang II stimulation enriched pathways related to immune chemotaxis, extracellular matrix (ECM) organization, complement and coagulation systems, platelet function, NF-κB signaling, and T cell activation-effects that were diminished in CCL5-deficient mice under Ang II treatment (Figure 2C and Figure S6A, B). Conversely, both saline-treated WT and CCL5 KO Ang II groups displayed enrichment in metabolic pathways governing carbohydrates, amino acids, and lipid metabolism (Figure S6C, D). Further validation through KEGG and GO term analyses corroborated that Ang II elevated expression of pro-inflammatory genes, ECM regulators, platelet activators, NF-κB, and TGF-β signaling components, while repressing metabolic genes. These effects were substantially reversed in CCL5 KO mice (Figure 2D-G, S6E, F). To further elucidate the role of CCL5 in Ang II-activated biological processes and signaling pathways, correlation analysis was performed on 32 heart RNA-sequencing datasets from the GEO database (GSE261273 and GSE261275) 28. *Ccl5* gene expression showed a strong positive correlation with the signature genes involved in platelet activation, NF-κB, and TGF-beta signaling pathways (Figure 2H-J). Additionally, gene set variation analysis (GSVA) scores of these pathways also showed a robust positive correlation with *Ccl5* gene expression (Figure 2K). Collectively, these results confirm CCL5 deficiency protects from hypertensive cardiac fibrosis through reduced ECM deposition and suggest that the underlying mechanism involves the inhibition of platelet activation, TGF-beta signaling, and NF-κB signaling pathways.

### CCL5 deficiency reduces platelet activation to attenuate Ang II-promoted cardiac remodeling

**Figure 3:**
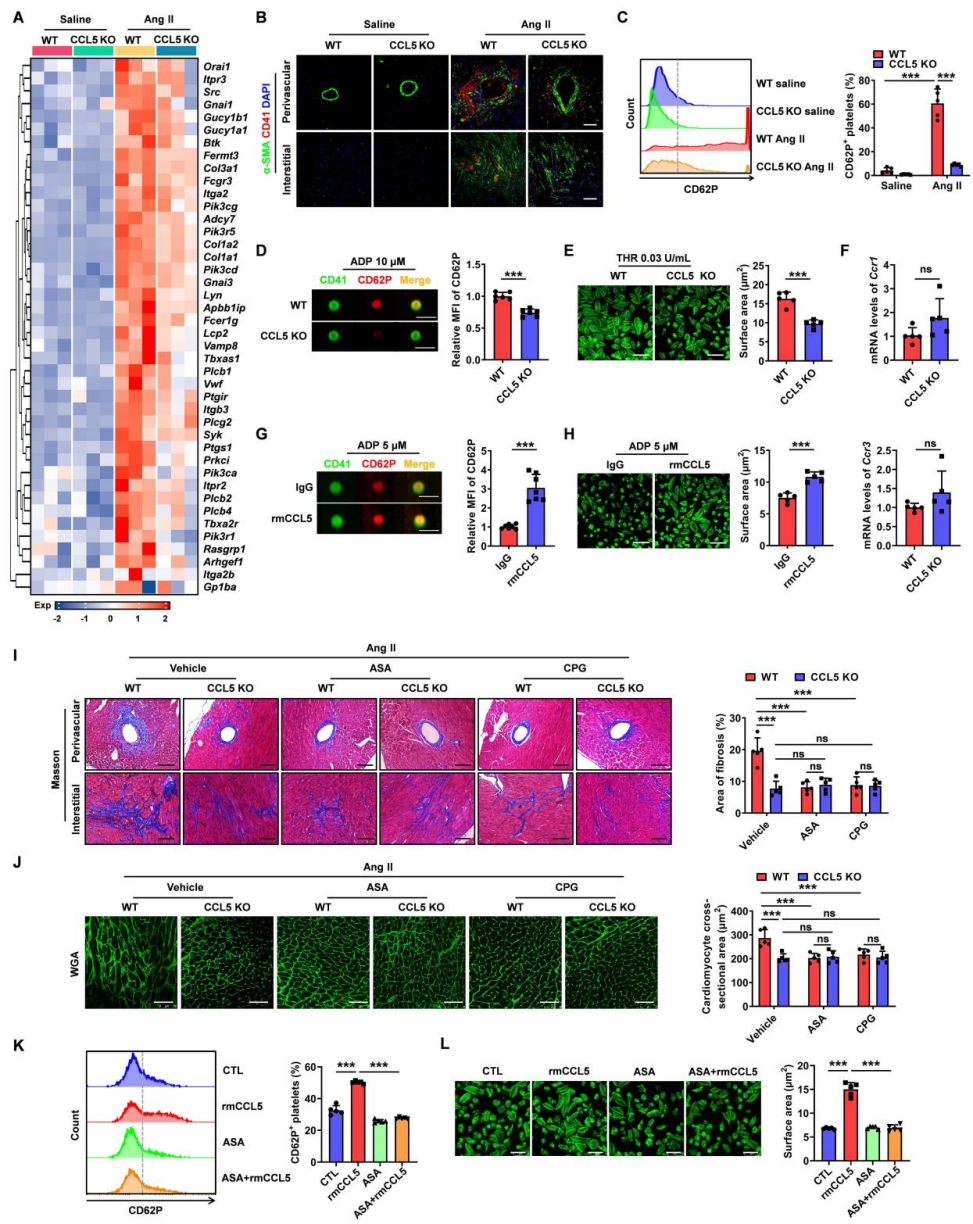
** CCL5 deficiency reduces platelet activation to attenuate Ang II-promoted cardiac remodeling.** WT and CCL5 KO mice were treated with saline or Ang II for 7 days. **A** Expression matrix data of platelet-associated genes in heart tissues, as detected by RNA sequencing (n = 3). **B** Immunofluorescence of platelets (red) and myofibroblasts (green) in the heart (n = 5; Scale bar: 50 μm). **C** Flow cytometry analysis of CD62P expression on circulating platelets in blood (n = 5). **D** Washed platelets from WT and CCL5 KO mice were activated by ADP (10 μM) for 10 minutes *in vitro*. The expression of CD62P was assessed by imaging flow cytometry (n = 6; Scale bar: 7 μm). **E** Washed platelets isolated from WT and CCL5 KO mice were stimulated with thrombin (THR, 0.03 U/mL) for 10 min prior to spreading on fibrinogen-coated wells for 2 h. Representative images of platelet spreading and quantification of spread area (μm^2^) are shown (n = 5; Scale bar: 10 μm). **F** Expression of *Ccr1* and *Ccr3* genes in platelets (n = 5). WT platelets stimulated by recombinant CCL5 (rmCCL5, 100 ng/mL) in the presence of ADP (10 μM) for 10 minutes *in vitro.*** G** Imaging flow cytometry analysis of CD62P expression (n = 7; Scale bar: 7 μm). **H** Representative images of platelet spreading and quantification of spread area (μm^2^) (n = 5; Scale bar: 10 μm). WT and CCL5 KO mice were infused with saline or Ang II for 7 days and treated with or without aspirin (ASA, 0.4 mg/mL) or clopidogrel (CPG, 0.15 mg/mL) in their drinking water (n = 5 per group). **I** Masson's trichrome staining of myocardial fibrosis (Scale bar: 100 μm). **J** WGA staining illustrating cardiomyocyte cross-sectional area (Scale bar: 50 μm). Washed platelets from WT mice were pretreated with or without ASA (2 mM) for 15 min, then stimulated with rmCCL5 (100 ng/mL) in the presence of ADP (10 μM) for 10 min *in vitro*. **K** The percentage of CD62P-positive platelets was determined by flow cytometry analysis (n = 5). **L** Representative images of platelet spreading and quantification of platelet spread areas (μm^2^) (n = 5; Scale bar: 8 μm). Data are presented as mean ± standard deviation, with n representing the number of animals. * P < 0.05, ** P < 0.01, *** P < 0.001, and NS indicating no significance.

RNA sequencing revealed that CCL5 deficiency dampened Ang II-induced platelet-associated gene expression, likely due to reduced platelet infiltration or diminished platelet activation (Figure 3A). To further identify the impact of CCL5 on platelet deposition in hypertensive cardiac remodeling, immunofluorescence for CD41 was performed on heart tissues. Ang II infusion significantly increased platelet accumulation, with the accumulated platelets (CD41) predominantly localized with the fibrotic areas (α-SMA) in WT Ang II hearts. This platelet accumulation was markedly reduced in CCL5 KO hearts (Figure 3B). Platelet activation was assessed by measuring the release of P-selectin from platelet α-granules, indicated by surface CD62P expression. CCL5 KO significantly reduced CD62P-positive platelets in peripheral blood (Figure 3C). Additionally, imaging flow cytometry of platelets stimulated with ADP *in vitro* revealed a decrease in α-granule release in the absence of CCL5 (Figure 3D). Platelet spreading upon thrombin stimulation was also reduced in CCL5 KO mice (Figure 3E). RT-PCR analysis showed that *Ccr1* and *Ccr3* gene expression in washed platelets was unaffected by CCL5 deficiency (Figure 3F). The interactions of CCL5 with CCR1 and CCR3 were confirmed by molecular docking analysis (Figure S7). Furthermore, recombinant CCL5 (rmCCL5) directly enhanced platelet α-granule release and spreading (Figure 3G, H). These results suggest that CCL5 deficiency not only reduces platelet accumulation in Ang II-infused hearts but also directly suppresses platelet activation, without altering the expression of CCL5 receptors on platelets.

To assess whether platelet activation inhibition is critical for the protection of CCL5 deficiency in cardiac injury, platelet inhibitors, including aspirin (ASA) and clopidogrel (CPG), were administered to WT and CCL5 KO mice infused with Ang II. As anticipated, both ASA and CPG significantly mitigated cardiac fibrosis and hypertrophy in Ang II-infused WT mice (Figure 3I, J). However, no additional protective effects were observed in CCL5 KO mice infused with Ang II (Figure 3I, J). The direct effects of ASA on rmCCL5-stimulated platelets were then evaluated. ASA was found to completely abolish the rmCCL5-induced upregulation of platelet surface CD62P expression and platelet spreading (Figure 3K, L). These results confirm that CCL5 modulates platelet activation to promote hypertensive cardiac remodeling.

### CCL5 deficiency attenuates Ang II-promoted cardiac inflammation

**Figure 4:**
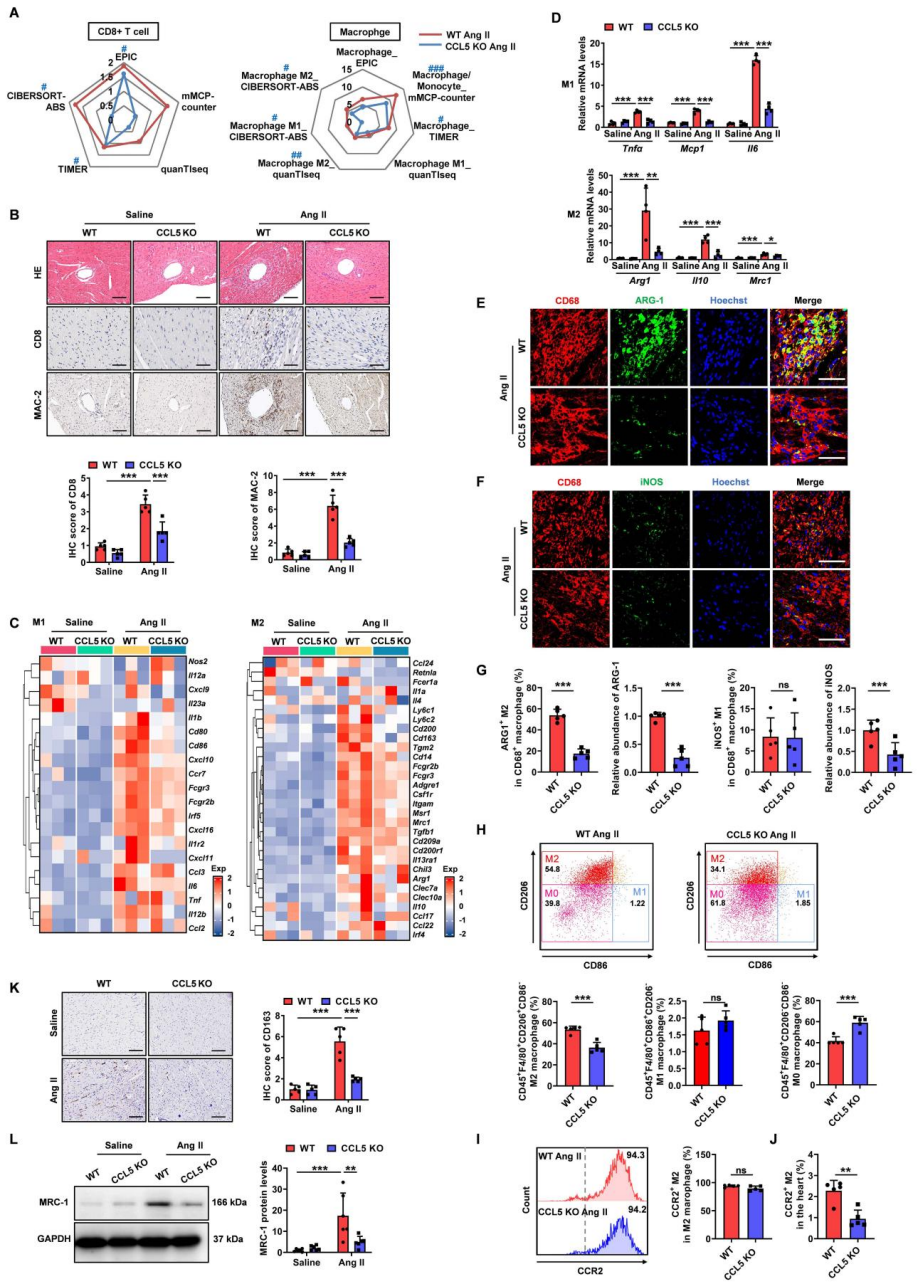
** CCL5 deficiency attenuates Ang II-promoted cardiac inflammation.** WT and CCL5 KO mice were subjected to a 7-day infusion of saline or Ang II. **A** Radar plot illustrating the extent of CD8^+^ T cells and macrophages, including M1 and M2 phenotypes, comparing CCL5 KO Ang II hearts to WT Ang II hearts using five algorithms (n = 3; # P adj < 0.05, ## P adj < 0.01, ### P adj < 0.001 represents the CCL5 KO Ang II group compared to the WT Ang II group, with downregulation in blue). **B** HE staining and immunohistochemical staining of CD8^+^ T cells and macrophages (MAC-2 positive) (n = 5; Scale bar: 100 μm for HE and MAC-2, 50 μm for CD8). **C** Enrichment map of genes related to M1 and M2 macrophage phenotypes detected by RNA-sequencing (n = 3). **D** RT-PCR detection of M1 (*Tnfα*, *Mcp1*, and *Il6*) and M2 (*Arg1*, *Il10*, and *Mrc1*) macrophage markers (n = 4). Immunofluorescence of CD68 (red), ARG-1 (green) (**E**) and iNOS (green) (**F**) in the heart (n = 5; Scale bar: 50 μm). **G** The abundance and proportion of ARG-1^+^ M2 and iNOS^+^ M1 macrophage were quantified. **H** Flow cytometry analysis of M2 macrophage (CD45^+^F4/80^+^CD206^+^CD86^-^), M1 macrophage (CD45^+^F4/80^+^CD86^+^CD206^-^), and M0 macrophage (CD45^+^F4/80^+^CD206^-^CD86^-^) in the hearts after Ang II infusion. The proportion relative to cardiac macrophages was quantified (n = 5). Flow cytometry analysis of CCR2^+^ M2 macrophage in the heart. The proportion relative to total M2 macrophages (**I**) and cardiac cells (**J**) were quantified (n =5). **K** Immunohistochemical staining of CD163 in the hearts. (n = 5; Scale bar: 100 μm). **L** Western blotting analysis of MRC-1 protein levels in the hearts (n = 6). Data are presented as the mean ± standard deviation, with n representing the number of animals. * P < 0.05, ** P < 0.01, *** P < 0.001.

To investigate the impact of CCL5 deficiency on Ang II-promoted cardiac inflammation, five deconvolution algorithms were employed to analyze RNA-sequencing data, revealing the immune cell infiltration profile in WT and CCL5 KO hearts with or without Ang II stimulation. The accumulation of various immune cell types observed in Ang II-infused WT hearts was notably reduced by CCL5 KO (Figure S8A), including memory B cells, macrophages/monocytes, cancer-associated fibroblasts, CD8^+^ T cells, myeloid dendritic cells, M2 macrophages, regulatory T cells, and NK cells. Among these, the distribution of CD8+ T cells (EPIC, TIMER, and CIBERSORT-ABS), macrophages (mMCPCounter and TIMER), and M2 macrophages (QUANTISEQ and CIBERSORT-ABS) was confirmed by two or more algorithms (Figure 4A and Figure S8A, B). Pathological staining corroborated the reduction of CD8^+^ T cells and macrophages in Ang II-infused CCL5 KO hearts (Figure 4B). RNA-sequencing revealed the downregulation of gene markers for pro-inflammatory M1 and pro-fibrotic M2 macrophages in Ang II-infused CCL5 KO hearts compared to WT hearts, a finding confirmed by RT-PCR (Figure 4C, D). Compared to M1 macrophages, M2 macrophages exhibit a more pronounced capacity to stimulate myofibroblast differentiation. This was demonstrated by the observation that supernatants from M2 macrophages significantly increased the protein levels of α-SMA and the gene expression of *Sm22*, *Col1a1*, and *Col3a1* in cardiac fibroblasts, relative to supernatants from M1 macrophages (Figure S9). The majority of macrophages in Ang II-infused WT hearts highly expressed the M2 macrophage marker ARG-1, while only a small fraction exhibited high expression of the M1 macrophage marker iNOS, indicating a more critical role for M2 macrophages in Ang II-induced cardiac remodeling and inflammation (Figure 4E-G). CCL5 KO significantly attenuated the Ang II-induced increase in both the abundance and proportion of M2 macrophages in the heart (Figure 4E and G). Although the quantity of iNOS-expressing M1 macrophages in the heart was reduced in CCL5 KO mice compared to WT mice in response to Ang II, the proportion of M1 macrophages remained comparable between the two groups (Figure 4F and G). Cardiac macrophage subpopulations were further investigated using flow cytometic analysis. The overall abundance of macrophages within total cardiac cells was significantly reduced in CCL5 KO mice (Figure S10).

Among cardiac macrophages in Ang II-infused WT hearts, the predominant subset was M2 macrophages (CD206^+^CD86^-^), the proportion of which was decreased in CCL5 KO hearts (Figure 4H). In contrast, the percentage of M1 macrophages (CD86^+^CD206^-^) showed no significant change, while that of M0 macrophages (CD206^-^CD86^-^) was increased in CCL5 KO hearts (Figure 4H). In both WT and CCL5 KO hearts, M2 macrophages were predominantly CCR2⁺, with no significant difference in the CCR2⁺ proportion within the M2 subpopulation (Figure 4I). However, the relative proportion of CCR2⁺ M2 macrophages among total cardiac cells was reduced in CCL5 KO mice compared to WT mice (Figure 4J). These findings suggest that CCL5 modulates cardiac M2 macrophage abundance primarily through regulating the recruitment of circulating monocytes. Additionally, immunohistochemical staining showed that the Ang II-induced CD163-positive M2 macrophages in WT hearts was significantly diminished in CCL5 KO hearts (Figure 4K). Western blotting further confirmed the reduction in M2 macrophage mannose receptor 1 (MRC-1) expression in CCL5 KO hearts in response to Ang II (Figure 4L). These results indicate that CCL5 deficiency alleviates Ang II-promoted cardiac inflammation, specifically by reducing the accumulation of CD8^+^ T cells and macrophages, particularly pro-fibrotic M2 macrophages.

### CCL5-deficient platelets attenuate Ang II-promoted cardiac remodeling by suppressing M2 macrophage polarization

**Figure 5:**
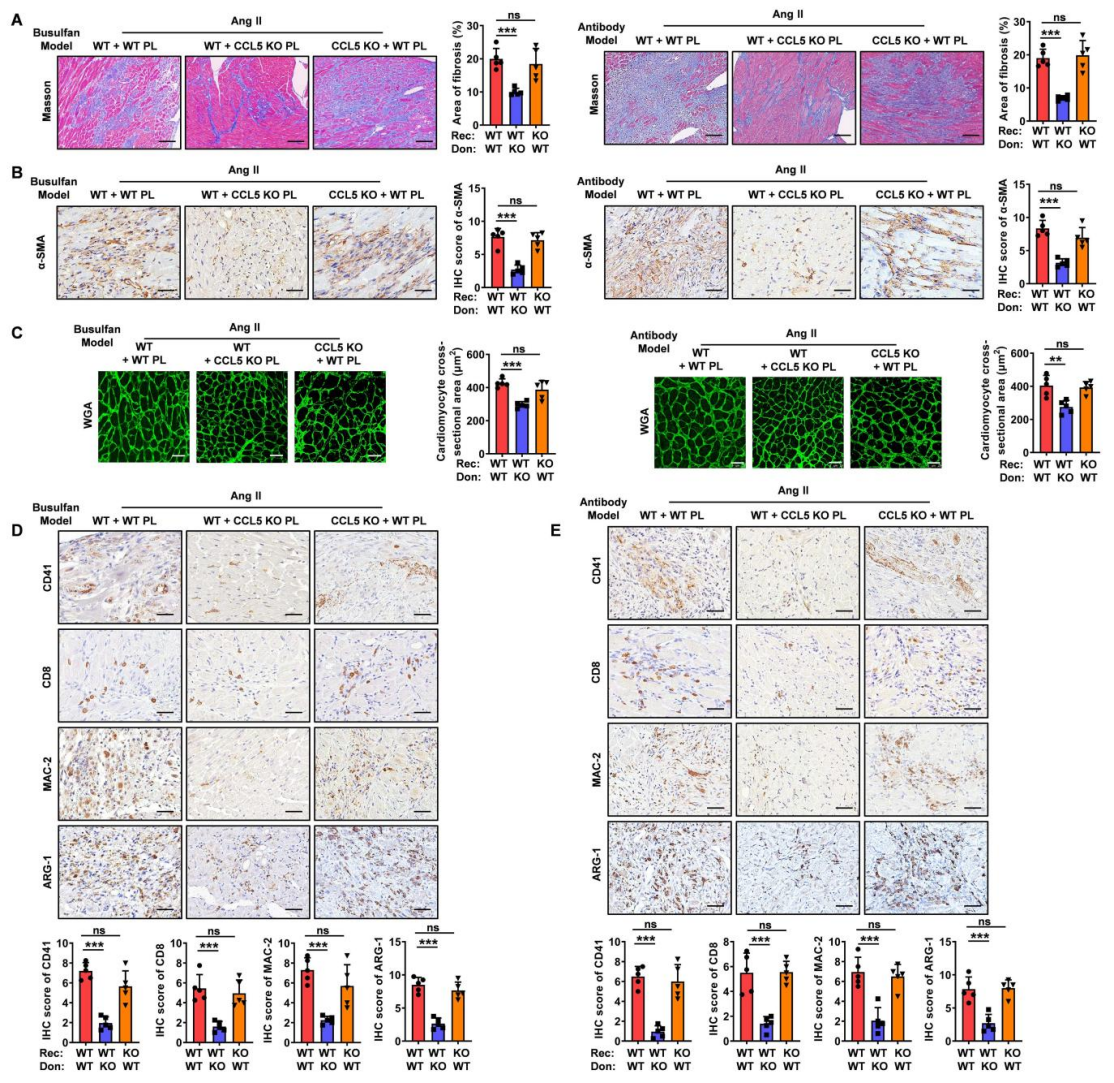
** CCL5-deficient platelets attenuate Ang II-promoted cardiac remodeling by suppressing M2 macrophage polarization.** Two distinct platelet depletion models: one induced by the chemotherapeutic agent busulfan (busulfan model) and another by a specific anti-platelet antibody (antibody model) were established. The platelet-depleted mice were reconstituted with platelets derived from either WT or CCL5 KO mice prior to Ang II infusion. The experimental groups were designated as follows (n = 5 per group): WT + WT PL (WT mice reconstituted with WT platelets), WT + CCL5 KO PL (WT mice reconstituted with CCL5 KO platelets), and CCL5 KO + WT PL (CCL5 KO mice reconstituted with WT platelets). **A** Masson's trichrome staining of myocardial fibrosis (Scale bar: 100 μm). **B** Immunohistochemistry of α-SMA (Scale bar: 200 μm). **C** WGA staining showed cardiomyocyte cross-sectional area (Scale bar: 25 μm). **D-E** Immunohistochemical staining for platelets (CD41), CD8^+^ T cells, macrophages (MAC-2), and M2 macrophages (ARG-1) in heart tissue (Scale bar: 200 μm). Data are presented as mean ± standard deviation, with n representing the number of animals. * P < 0.05, ** P < 0.01, *** P < 0.001, NS indicating no significance.

Numerous studies have demonstrated that platelets play a critical role as both initiators and amplifiers of the inflammatory response, actively engaging in immune cell recruitment and activation 6. In this study, impaired platelet function and reduced immune cell infiltration were observed in Ang II-infused CCL5 KO mice compared to WT controls. To ascertain whether the suppressed platelet function stemming from CCL5 deficiency contributed to the attenuation of cardiac remodeling and inflammation, two distinct platelet depletion/reconstitution models were employed. In the first model, recipient mice underwent platelet depletion *via* administration of the chemotherapeutic agent busulfan. In the second, platelet depletion was achieved through the use of a specific anti-platelet antibody. Subsequently, these platelet-depleted mice were reconstituted with either WT or CCL5 KO platelets. Platelet counts were assessed before depletion, after depletion, and after reconstitution to confirm successful depletion and reconstitution (Figure S11). Subsequently, hypertension was induced by 7-day Ang II infusion. In both platelet depletion/reconstitution models, WT mice reconstituted with CCL5 KO platelets exhibited reduced cardiac fibrosis, myofibroblast differentiation and cardiomyocyte hypertrophy than those reconstituted with WT platelets (Figure 5A-C). Histological analysis confirmed fewer CD41-positive platelets in the hearts of WT mice receiving CCL5 KO platelets compared to those receiving WT platelets (Figure 5D, E). Additionally, reconstitution with CCL5 KO platelets resulted in a significant reduction in the Ang II-induced infiltration of CD8^+^ T cells, macrophages, and M2 macrophages in the hearts of WT mice (Figure 5D, E). The reduction in fibrosis, inflammation, and M2 macrophage gene expression in CCL5 KO platelet-reconstituted WT mice in busulfan-induced platelets depletion model was further validated by RT-PCR (Figure S12). Conversely, reconstitution of CCL5 KO mice with WT platelets resulted in similar pathological changes in response to Ang II, comparable to WT mice reconstituted with WT platelets, across both busulfan and antibody-induced platelet depletion models (Figure 5A-E). This suggests that CCL5 deficiency in non-platelet cell types does not reverse Ang II-promoted cardiac remodeling and inflammation, highlighting the essential role of impaired platelet function resulting from CCL5 deficiency in attenuating these processes.

**Figure 6:**
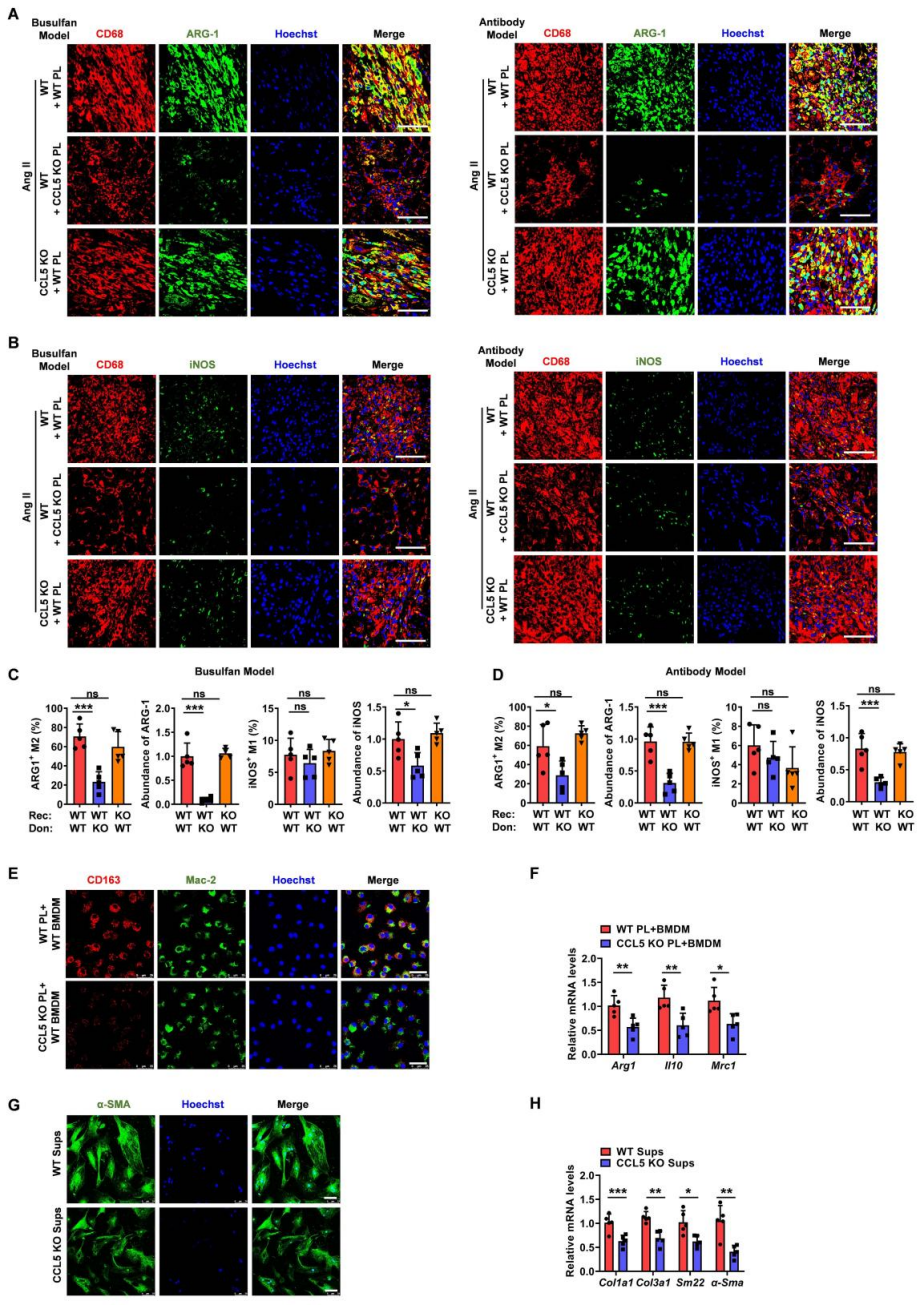
** CCL5-deficient platelets attenuate Ang II-induced M2 macrophage polarization.** Recipient mice underwent platelet depletion *via* either busulfan-induced chemotherapy or administration of the anti-platelet antibody. Subsequently, these mice were reconstituted with platelets derived from either WT or CCL5 KO mice before initiating Ang II infusion. The experimental groups were designated as follows (n = 5 per group): WT + WT PL (WT mice reconstituted with WT platelets), WT + CCL5 KO PL (WT mice reconstituted with CCL5 KO platelets), and CCL5 KO + WT PL (CCL5 KO mice reconstituted with WT platelets). Immunofluorescence of CD68 (red), ARG-1 (green) (**A**) and iNOS (green) (**B**) in the heart (Scale bar: 50 μm). **C, D** The abundance and proportion of ARG-1^+^ M2 and iNOS^+^ M1 macrophage were quantified. Bone marrow-derived macrophages (BMDM) were co-cultured with ADP-activated WT or CCL5 KO platelets in the presence of Ang II (100 nM) for 48 hours. Macrophages were analyzed for M2 markers, and the supernatants were collected for myofibroblast stimulation. **E** Immunofluorescence of CD163 on macrophages (MAC-2) (n = 3; Scale bar: 25 μm). **F** Gene expression of M2 markers (*Arg1*, *Il10*, and *Mrc1*) in macrophages (n = 5). **G** Immunofluorescence of myofibroblasts with α-SMA (n = 3; Scale bar: 75 μm). **H** Gene expression of myofibroblast markers (*Col1a1*, *Col3a1*, *Sm22*, and *α-Sma*) detected by RT-PCR (n = 5). Data are presented as mean ± standard deviation, with n representing the number of animals. * P < 0.05, ** P < 0.01, *** P < 0.001, NS indicating no significance.

To further examine the role of platelet CCL5 deficiency on M2 macrophage polarization within the heart, we assessed cardiac macrophage composition. Immunofluorescence staining revealed that reconstitution with CCL5 KO platelets significantly decreased the proportion of M2 macrophages in Ang II-infused WT hearts, while exerting no significant effect on the proportion of M1 macrophages (Figure 6A-D). However, reconstitution of CCL5 KO mice with WT platelets exhibited similar cardiac macrophage composition in response to Ang II, as compared to WT mice reconstituted with WT platelets, in both the busulfan and antibody-induced platelet depletion mouse models (Figure 6A-D).

To examine the direct impact of CCL5-activated platelets on M2 macrophage polarization, WT or CCL5 KO platelets were co-cultured with WT macrophages *in vitro*. In the presence of Ang II and ADP, macrophages co-cultured with CCL5 KO platelets exhibited a significant decrease in CD163 protein expression, as well as in the mRNA levels of *Arg1*, *Il10*, and *Mrc1*, compared to macrophages co-cultured with WT platelets (Figure 6E, F). To assess the influence of M2 macrophages suppression by CCL5-deficient platelets on subsequent myofibroblast differentiation, fibroblasts were cultured with cell-free supernatants from macrophages co-cultured with either WT or CCL5 KO platelets. Fibroblasts exposed to supernatants from macrophages co-cultured with CCL5 KO platelets showed a marked reduction in α-SMA protein levels, along with decreased mRNA expression of *Col1a1*, *Col3a1*, *Sm22*, and *α-Sma*, compared to those stimulated with supernatants from macrophages co-cultured with WT platelets (Figure 6G, H). To address the potential for macrophage-intrinsic CCL5 deficiency to confound the observed polarization effects, bone marrow-derived macrophages (BMDM) from WT and CCL5 KO mice were stimulated with Ang II *in vitro*. Gene expression analysis revealed no significant differences between the two groups in the expression of M1 macrophage markers (*Mcp1*, *Tnfa*, *Il6*, *Inos*, *Cd80,* and *Cd86*) or M2 macrophage markers (*Tgfb1*, *Il10*, *Arg1*, *Mrc1*, and *Ym1*) in response to Ang II stimulation (Figure S13A, B). Furthermore, supernatants collected from these stimulated macrophages exhibited comparable effects on myofibroblast differentiation (Figure S13C, D). This indicates that CCL5 deficiency within macrophages does not directly influence myofibroblast activation in this context. These results suggest that CCL5-mediated platelet activation plays a pivotal role in driving M2 macrophage polarization, which in turn promotes subsequent myofibroblast differentiation.

### CCL5 deficiency reduces platelet-derived TGF-β1 in cardiac remodeling

**Figure 7:**
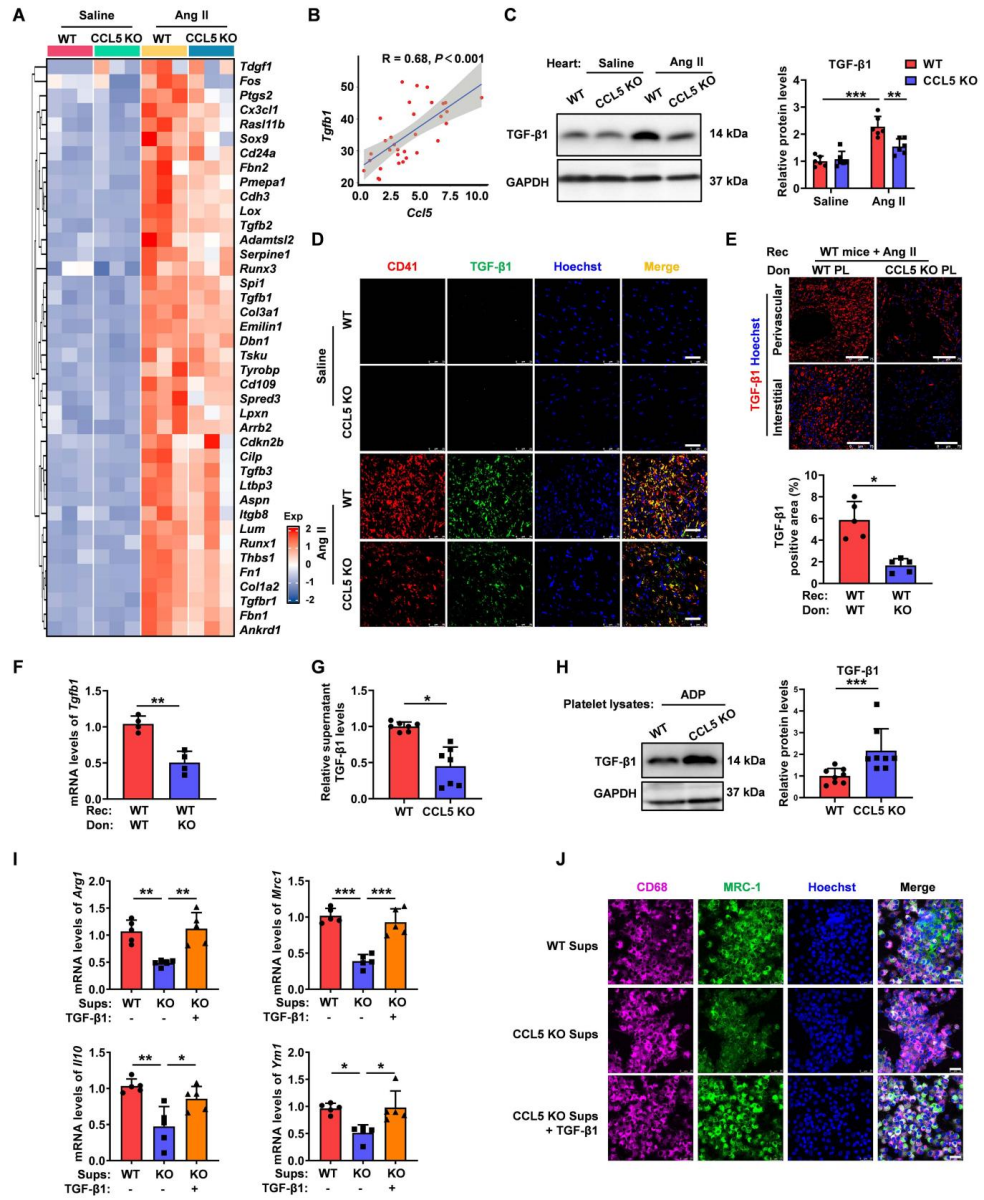
** CCL5 deficiency reduces platelet-derived TGF-β1 in cardiac remodeling.** WT and CCL5 KO mice were subjected to a 7-day infusion of saline or Ang II. **A** Gene expression of the TGF-β signaling pathway in the hearts, detected by RNA sequencing (n = 3). **B** Correlation between *Ccl5* and *Tgfb1* gene expression in hearts following a 7-day infusion of either Ang II or vehicle, using GEO datasets (n = 32). **C** TGF-β1 protein levels in the heart analyzed by Western blotting (n = 6). **D** Immunofluorescence of platelets (CD41, red) and TGF-β1 (green) in the heart (n = 5; Scale bar: 25 μm). WT mice were platelet depleted by busulfan, followed by reconstitution with platelets derived from either WT or CCL5 KO mice prior to Ang II infusion. **E** Immunofluorescence of TGF-β1 (red) in the heart (n = 5; Scale bar: 75 μm). **F** mRNA levels of *Tgfb1* in the hearts detected by RT-PCR (n = 4). Washed platelets from WT and CCL5 KO mice were stimulated with ADP (10 μM) for 10 minutes. TGF-β1 levels were detected in the supernatants by ELISA (**G**) (n = 7) and in platelet lysates by Western blotting (**H**) (n = 8). Bone marrow-derived macrophages (BMDM) were co-cultured with supernatants from ADP plus Ang II-activated WT or CCL5 KO platelets with or without TGF-β1 (20 ng/mL) for 48 hours. Macrophages were analyzed for M2 markers. **I** Gene expression of M2 markers (*Arg1*,* Il10*, *Mrc1* and *Ym1*) in macrophages (n = 5). **J** Immunofluorescence of MRC-1 on macrophages (CD68) (n = 5; Scale bar: 25 μm). Data are presented as mean ± standard deviation, with n representing the number of animals. * P < 0.05, ** P < 0.01, *** P < 0.001.

Platelets contain approximately 40 times more TGF-β1 than other cell types, which is released rapidly upon platelet activation 32. Compared to BMDMs, splenocytes, cardiac fibroblast, cardiomyocytes and endothelial cells, only platelets rapidly released substantial amounts of TGF-β1 as soon as 10 minutes following ADP plus Ang II stimulation, with this effect persisting for up to 24 hours (Figure S14A). Upon 3 days of Ang II treatment, platelets were identified as the major contributor to cardiac TGF-β1 during the initial phase (Figure S14B). These demonstrate the crucial role of platelet-derived TGF-β1 in triggering Ang II-related pathological responses. This study investigated whether CCL5 deficiency could alleviate Ang II-promoted cardiac remodeling by suppressing platelet-derived TGF-β1. Following Ang II infusion, transcriptional upregulation of key components within the TGF-β pathway was observed in WT mice, an effect that was significantly attenuated in animals lacking CCL5 (Figure 7A). The gene expression of cardiac *Ccl5* showed a strong positive correlation with *Tgfb1* in Ang II-infused mice (Figure 7B). Additionally, the hearts of CCL5 KO mice exhibited a significant attenuation in the Ang II-induced elevation of TGF-β1 protein levels relative to WT controls (Figure 7C). Immunofluorescence analysis further verified that following Ang II stimulation, cardiac TGF-β1 expression and platelet infiltration (CD41-positive) were decreased in CCL5 KO mice, and TGF-β1 was mainly found to co-localize with platelets (Figure 7D). In the platelet depletion/reconstitution model, WT mice that received CCL5 KO platelet transplants demonstrated markedly lower cardiac TGF-β1, both in protein content and mRNA expression, relative to those receiving WT platelets (Figure 7E, F). CCL5 KO platelets released less TGF-β1 in the supernatant following ADP stimulation, compared to WT platelets (Figure 7G). Interestingly, more TGF-β1 was retained within CCL5 KO platelets after ADP stimulation (Figure 7H). Furthermore, recombinant TGF-β1 reversed the suppression of M2 markers in macrophages treated with supernatants from CCL5 KO platelets, as evidenced by upregulated mRNA expression of *Arg1*, *Il10*, *Mrc1*, and *Ym1*, along with increased protein levels of MRC-1 (Figure 7I, J). These results suggest that platelets are a major source of TGF-β1 in hypertensive cardiac remodeling and that CCL5 is essential for elevating platelet-derived TGF-β1 to promote M2 polarization.

### CCL5 deficiency dampens NF-κB signaling activation in the heart and platelet

**Figure 8:**
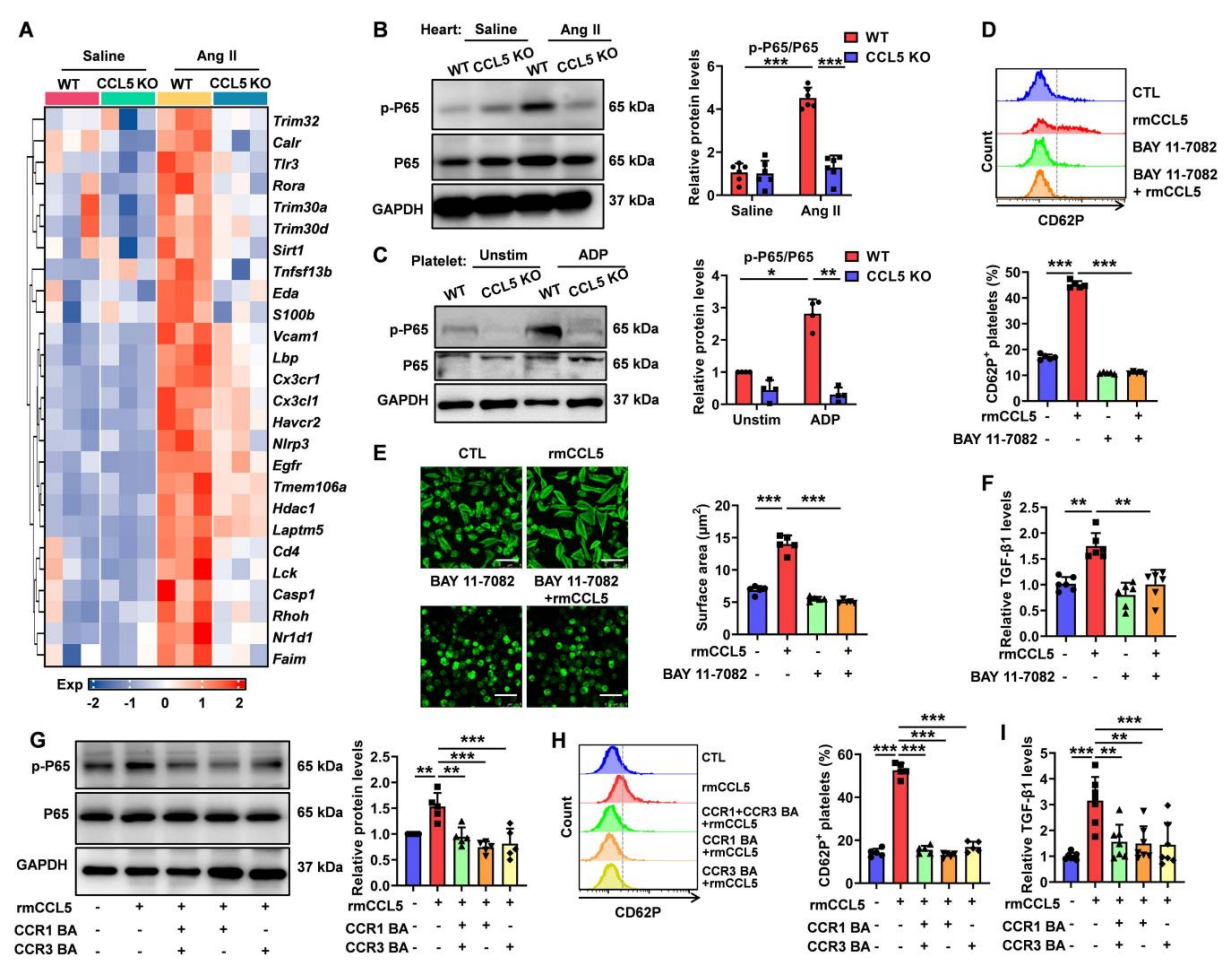
** CCL5 deficiency dampens NF-κB signaling activation in the heart and platelets.** WT and CCL5 KO mice were infused with saline or Ang II for 7 days. **A** Heatmap of NF-κB signaling pathway gene expression in hearts, analyzed by RNA sequencing (n = 3). **B** NF-κB activation in the heart, reflected by the p-P65/P65 protein ratio, was detected by Western blotting analysis (n = 6). **C** Washed platelets from WT and CCL5 KO mice were stimulated with or without ADP (10 μM) for 10 minutes, and NF-κB activation was assessed by Western blotting (n = 4). WT platelets were pretreated with or without the NF-κB inhibitor BAY 11-7082 (1 μM) for 15 minutes, followed by stimulation with rmCCL5 (100 ng/mL) in the presence of ADP (5 μM) for 10 minutes *in vitro*. **D** The percentage of CD62P-positive platelets after rmCCL5 stimulated for 10 minutes was measured by flow cytometry (n = 5). **E** Representative images of platelet spreading on fibrinogen-coated wells at 37°C for 2 hours and quantification of spread area (μm^2^) (n = 5; Scale bar: 8 μm).** F** TGF-β1 levels in supernatants of platelets detected by ELISA (n = 6). WT platelets were pretreated with CCR1 and CCR3 blocking antibodies (CCR1 BA and CCR3 BA) alone or in combination for 15 minutes, followed by stimulation with rmCCL5 (100 ng/mL) in the presence of ADP (5 μM) for 5 minutes *in vitro*. **G** Western blotting analysis of NF-κB activation (n = 5). **H** The percentage of CD62P-positive platelets was measured by flow cytometry (n = 5). **I** TGF-β1 levels in supernatants were detected by ELISA (n = 7). Data are presented as mean ± standard deviation, with n representing the number of animals. * P < 0.05, ** P < 0.01, *** P < 0.001.

NF-κB signaling plays a critical role in cardiac inflammation and remodeling and is also known for its involvement in platelet-mediated immune inflammation 33, 34. To explore whether CCL5 promotes cardiac remodeling and platelet activation through NF-κB pathway enhancement, gene expression analysis was conducted. NF-κB signaling pathway genes showed upregulation in Ang II-treated WT hearts, which was notably reduced in CCL5 KO hearts (Figure 8A). Ang II promoted NF-κB activation reflected by increased phosphorylation of P65 in WT hearts, which was significantly lowered by CCL5 KO (Figure 8B). The direct effect of CCL5 on NF-κB signaling in platelets was also assessed. CCL5 KO markedly reduced ADP-stimulated phosphorylation of P65 (Figure 8C). Inhibition of NF-κB signaling with BAY 11-7082 efficiently abrogated the enhancing effects of rmCCL5 on platelet P-selectin release and on platelet spreading (Figure 8D, E). Furthermore, rmCCL5 stimulation led to increased TGF-β1 secretion in WT platelets, an effect abolished by NF-κB inhibition (Figure 8F). Within platelets, TGF-β1 is stored in α-granules, the secretion of which is linked to the actin cytoskeleton 35. To determine whether CCL5 promotes α-granule release and TGF-β1 secretion by triggering rapid actin cytoskeleton remodeling *via* the NF-κb signaling, the morphology and distribution of F-actin in platelets after 10 min of rmCCL5 stimulation was detected. Immunofluorescence revealed that F-actin polymerization increased upon rmCCL5 stimulation and reduced by NF-κB inhibitor (Figure S15).

These suggest that the NF-κB pathway may regulate α-granules release and TGF-β1 secretion by modulating the actin cytoskeleton. Macrophages treated with WT platelet supernatant in the presence of the NF-κB inhibitor BAY 11-7082 exhibited downregulated expression of the M2 marker genes *Mrc1* and *Ym1* along with reduced MRC-1 protein levels, whereas the expression of *Arg1* and *Il10* remained unaffected (Figure S16).

While the expression of CCL5 receptors CCR1 and CCR3 on platelets has been confirmed, their specific biological functions in this context remain underexplored 15. To elucidate the functional receptors responsible for CCL5-mediated effects on platelets, we utilized blocking antibodies targeting CCR1 and CCR3. Individual or combined application of these antibodies effectively inhibited rmCCL5-stimulated platelet p65 phosphorylation, α-granule secretion, and TGF-β1 secretion (Figure 8G-I). Notably, no statistically significant difference in inhibitory efficacy was observed between the individual and combined antibody treatments (Figure 8G-I). These results suggest that CCL5 promotes platelet activation and TGF-β1 secretion *via* the NF-κB pathway, mediated by both CCR1 and CCR3. This mechanism further contributes to hypertensive cardiac remodeling.

### Biological pathway networks modulated by CCL5 during hypertensive cardiac remodeling

**Figure 9:**
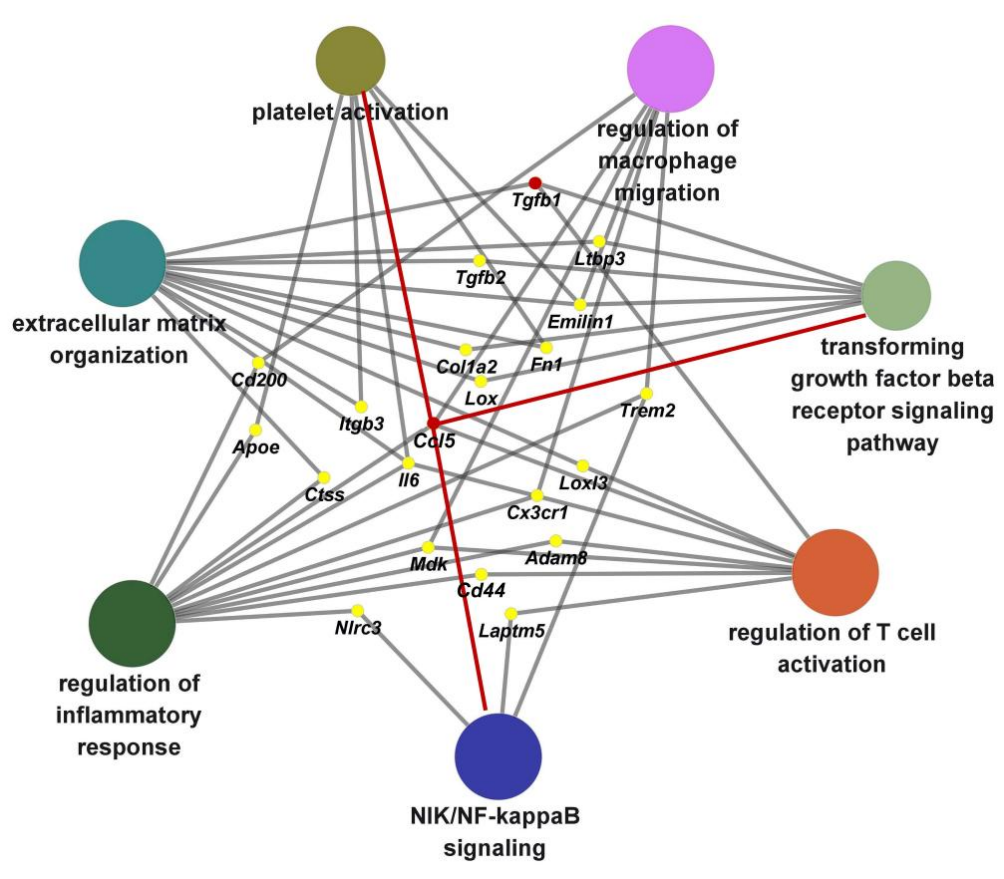
** Biological pathway networks modulated by CCL5 during hypertensive cardiac remodeling.** Functional enrichment analysis using ClueGO was performed on CCL5-dependent genes from Ang II-infused hearts, revealing functionally clustered Gene Ontology (GO) biological processes categorized by color. Key enriched terms included inflammatory response regulation, macrophage and T cell activation, platelet function, NIK/NF-κB and TGF-β receptor signaling, and extracellular matrix organization. Nodes correspond to significantly enriched GO terms, with edges indicating shared genes between processes. Gray edges reflect connections established automatically by ClueGO, while red edges highlight novel interactions identified in this study.

To delineate the principal biological pathways through which CCL5 contributes to hypertension-induced cardiac remodeling, we performed a ClueGO-based functional annotation of CCL5-regulated genes. This analysis identified significant enrichment in multiple key processes, including immune activation, macrophage and T cell stimulation, platelet function, NIK/NF-κB signal transduction, TGF-β receptor signaling, and extracellular matrix (ECM) assembly (Figure 9). Among these, molecules implicated in two or more functional clusters were *Ccl5*, *Tgfb1*, *Tgfb2*, *Ltbp3*, *Emilin1*, *Fn1*, *Col1a2*, *Lox*, *Cd200*, *Itgb3*, *Trem2*, *Apoe*, *Ctss*, *Il6*, *Loxl3*, *Cx3cr1*, *Mdk*, *Cd44*, *Adam8*, *Nlrc3,* and *Laptm5* (Figure 9). Collectively, these results demonstrate that CCL5 promotes ECM deposition by orchestrating inflammatory responses involving platelets, macrophages, and T cells, primarily through activation of NF-κB and TGF-β signaling pathways in the hypertensive heart.

## Discussion

Hypertensive cardiac remodeling markedly increases the risk of cardiovascular complications and is associated with adverse clinical outcomes. However, current treatments for hypertensive cardiac remodeling are limited and often associated with considerable side effects 36. Inflammation is a key driver in the onset of hypertension-induced damage to end organs. Our findings reveal an essential function of CCL5 in cardiac remodeling triggered by Ang II. CCL5 promotes platelet activation, facilitating cardiac inflammatory cascades and M2 macrophage polarization. Mechanistically, CCL5 activates NF-κB signaling *via* CCR1 and CCR3, leading to the upregulation of platelet-derived TGF-β1, which drives both M2 macrophage polarization and myofibroblast differentiation. Our results demonstrate that CCL5-mediated platelet activation contributes causally to the development of cardiac remodeling, thereby supporting the therapeutic potential of CCL5 inhibition in mitigating inflammatory processes associated with hypertensive heart remodeling.

CCL5 is elevated in various cardiovascular events, including hypertension, where it triggers the pathogenesis of cardiovascular dysfunction and renal injury 17, 37. During pulmonary arterial hypertension, CCL5 expression was associated with CD45^+^ inflammatory cell infiltration into the pulmonary artery wall, and CCL5 deletion attenuated Sugen5416/hypoxia-induced pulmonary arterial hypertension and pulmonary vascular remodeling 38, 39. Similarly, both pharmacological inhibition and genetic deletion of CCL5 mitigated Ang II-promoted vascular dysfunction in hypertension models 17. In contrast, genetic deletion of CCL5 exacerbated kidney damage during Ang II-dependent hypertension 18. According to previous research, in vascular smooth muscle cells (VSMCs) of spontaneously hypertensive rats (SHRs), CCL5 expression is suppressed by Ang II through 12-lipoxygenase (12-LO)-dependent mechanisms 40. To elucidate the molecular mechanisms underlying the early inflammatory response in cardiac remodeling, we established a mouse model of Ang II-induced cardiac hypertrophy *via* 7-day Ang II infusion. This model exhibits a compensatory elevation in cardiac function accompanied by pathological features such as myocardial fibrosis, hypertrophic remodeling, and inflammatory infiltration 4, 19, 41, 42. Our study demonstrated that Ang II increased cardiac CCL5 expression and targeting CCL5 ameliorated Ang II-induced pathological cardiac remodeling, thereby ameliorating the early compensatory cardiac function elevation. In contrast to the chronic hypertension and vascular remodeling in SHRs, our model of Ang II-infused mice primarily induces acute cardiac injury and remodeling. The discrepancy in the regulation of CCL5 by Ang II may be attributed to differences in the disease models and/or the distinct cell types involved. These disparate findings suggest that CCL5 plays context-dependent roles in hypertension-induced end-organ injury, a topic that remains under active investigation.

Previous studies have established that CCL5 modulates inflammatory diseases primarily by influencing the biology of T lymphocytes, monocytes, macrophages, and neutrophils 5, 16. Increasing evidence highlights the inflammatory role of platelets in cardiovascular disease 43. The protective effect of platelet inhibition has been observed in TAC-induced cardiac inflammation and fibrosis 7. Platelets express CCL5 receptors, CCR1 and CCR3, and secrete substantial amounts of CCL5 upon activation 15. One study demonstrated that CCL5 induces rapid, dose-dependent Ca^2+^ responses in platelets and promotes platelet aggregation *via* a feedback mechanism involving ADP release and its subsequent binding to ADP receptors 15. In contrast, another study reported no effect of CCL5 on Ca^2+^ flux in washed platelets or platelet aggregation in either washed platelets or platelets in platelet-rich plasma (PRP) 14. Thus, the direct effects of CCL5 on platelet function remain controversial. While previous studies have mainly focused on platelet aggregation regulation by CCL5, the role of CCL5 in platelet inflammation and its pathological significance remain poorly understood. In the present study, CCL5 was identified as a master regulator of platelet inflammatory activation. Our findings show that CCL5 directly modulates platelet α-granule release and TGF-β1 secretion. Pharmacological platelet inhibition and platelet depletion/reconstitution experiments provide further evidence for the critical role of CCL5-mediated platelet function in promoting cardiac inflammation and remodeling during Ang II-induced hypertension.

Through direct cellular contacts as well as secretory factors such as cytokines and microvesicles, platelets are pivotal in mediating leukocyte recruitment and activation, thereby fundamentally contributing to the onset and advancement of inflammatory disorders 6. Consequently, the diminished immune cell infiltration within the cardiac tissue of CCL5 KO mice following Ang II infusion can be attributed to both a blunted platelet-mediated inflammatory response and a reduction in direct chemotaxis resulting from CCL5 deficiency. Given the established roles of CD8^+^ T cells and macrophages in the pathogenesis and progression of cardiac remodeling 44, 45, the observed decrease in these cell populations, stemming from impaired platelet activation, likely further contributed to the attenuation of cardiac inflammation. Platelets regulate macrophage function by modulating activation, polarization, and differentiation. One study demonstrated that platelets promote pro-inflammatory iNOS^+^ macrophage polarization and bacterial clearance, thereby increasing survival in septic mice 46. Additionally, platelets enhance NLRP3 inflammasome activation and boost IL-1β secretion in macrophages 47. Conversely, platelet-derived THBS1 promotes the cycling of M2 macrophages, which exhibit a pro-repair M2 phenotype and enhanced proliferative activity during renal injury repair 48. In bacterial-induced pneumonia, platelets are recruited to the lung alongside neutrophils and regulatory T cells, modulating macrophage polarization toward an anti-inflammatory phenotype during the resolution phase 49. These studies suggest that the role of platelets in macrophage polarization varies depending on the specific inflammatory disease context. Our present work demonstrated a pivotal role for CCL5-dependent platelet activation in promoting M2 polarization to facilitate cardiac remodeling during Ang II infusion. This was further confirmed by *in vitro* co-culture experiments showing the direct effects of CCL5/platelet interaction on M2 polarization in the presence of Ang II.

Previous study showed that platelet-derived TGF-β1 contributes to plasma levels of TGF-β1 and the pathological cardiac changes occurring in response to aortic constriction 8. Our study revealed platelet-derived TGF-β1 was the major source of TGF-β1 in Ang II-induced early cardiac remodeling. Platelet-derived TGF-β1 needs to be activated to interact with its receptors, and Ang II infusion created conditions with multiple latent TGF-β1 activators, including thrombospondin-1, matrix metalloproteinases, RGD (Arg-Gly-Asp)-binding integrins, and reactive oxygen species 50-53. Platelets can quickly release large amounts of TGF-β1. In contrast, in other cardiac cells, Ang II mainly regulated TGF-β1 expression through transcriptional control or by triggering phenotypic changes, which process was relatively slow and happened as a delayed response after platelet activation. Thus, platelets appear to be a key player in the pathological cardiac remodeling triggered by Ang II, potentially initiating a cascade of pro-inflammatory multi-cellular events. CCL5 KO reduced platelet activation, which may weaken the platelet-driven initiation and amplification of inflammatory responses These included decreased infiltration of macrophages and CD8⁺ T cells, reduced M2 macrophage polarization, less fibroblast proliferation, and diminished myocardial hypertrophy, thereby lowering TGF-β1 levels from these cells. Overall, suppression of platelet activation is pivotal to ameliorating cardiac remodeling in CCL5 KO mice through the general reduction of inflammation in the cardiac environment.

NF-κB signaling plays a key role in hypertensive cardiac remodeling and inflammation 33. Numerous studies have confirmed its involvement in platelet-mediated immune inflammation 34. However, the specific NF-κB signaling pathway in platelets is not fully understood. Our data demonstrated a positive correlation between *Ccl5* expression and NF-κB signaling gene expression. CCL5 KO downregulated NF-κB signaling activation in Ang II-infused hearts and ADP-stimulated platelets. Furthermore, inhibition of NF-κB signaling abolished rmCCL5-induced platelet α-granule release and TGF-β1 secretion. Within platelets, TGF-β1 is stored in α-granules, the secretion of which is linked to the actin cytoskeleton 35. Blocking NF-κB signaling impeded CCL5-induced rapid F-actin polymerization. This suggests that the NF-κB pathway may regulate TGF-β1 release from α-granules by modulating the actin cytoskeleton, although the precise molecular mechanisms require further elucidation. Furthermore, this study provides novel evidence of the biological functions of CCR1 and CCR3 receptors on platelets, indicating that CCL5 modulates NF-κB signaling through these receptors to promote platelet activation.

This study presents several limitations and suggests potential future directions. The findings from global CCL5 KO mice do not rule out the possible involvement of CCL5 produced by cells other than platelets in Ang II-promoted cardiac remodeling, including T cells, macrophages, endothelial cells, fibroblasts, smooth muscle cells, and cardiomyocytes. CCL5 released by these cells might contribute to disease development by modulating immune cell infiltration, angiogenesis, and VSMCs proliferation 5, 16, 54, 55. Although our platelet transfusion experiments have demonstrated the essential and unique role of platelet-derived CCL5, further research using cell-specific knockout mouse models is needed to clarify the distinct roles of CCL5 from various cell sources. CCL5 exerts its biological effects through various receptors, including CCR1, CCR3, and CCR5. However, these receptors can also be engaged by alternative ligands; thus, strategies that inhibit a single receptor may offer only a partial view of CCL5's broad spectrum of activities and cannot preclude compensatory effects from other chemokine signaling pathways. For instance, prior research has shown that CCR5 deficiency did not attenuate injury to the heart and kidneys following treatment with DOCA-salt in combination with Ang II 56. While our study showed that CCL5-deficient mice are protected from Ang II-promoted cardiac remodeling, this discrepancy may be attributed to differences in mouse strains and the severity of the hypertension model. Further investigation of CCL5 receptors in the context of Ang II-induced cardiac remodeling would be valuable and is an area for future research. While our analysis of blood pressure in mice utilized the tail-cuff method, and revealed no substantial differences in average systolic blood pressure between CCL5 KO and WT mice in response to Ang II, consistent with prior studies employing radiotelemetry 17, 18, we acknowledge the inherent limitations of this technique. The tail-cuff method, while providing an average blood pressure reading over several minutes, lacks the capacity to capture dynamic hemodynamic fluctuations occurring throughout the day or during unrestricted activity 57. To mitigate potential inaccuracies and ensure comparability between groups, we adhered to a standardized procedure for tail-cuff measurements, focusing on average blood pressure rather than transient variations. In contrast, implantable radiotelemetry offers continuous and direct blood pressure monitoring, enabling assessment of temporal changes, including diurnal variations in hemodynamics, in freely moving animals. However, radiotelemetry is an invasive procedure associated with significant mortality and morbidity, and its cost restricts widespread implementation. Furthermore, the extensive instrumentation involved may influence inflammation; therefore, assessment of immune cell infiltration into target organs, circulating cytokines, and local chemokine levels in separate, non-instrumented animals is often preferred 58. The primary objective of this study was to investigate the immune mechanisms underlying CCL5-mediated hypertensive cardiac remodeling, and blood pressure effects were not the focus of this immune-centric mechanism. Considering constraints in experimental conditions and the availability of transgenic mice, we employed the non-invasive tail-cuff method. Future investigations incorporating continuous radiotelemetry monitoring could offer more nuanced insights into the circadian profile and activity-related hemodynamic effects of CCL5 deletion in hypertensive mice.

## Conclusions

In summary, our findings identify CCL5 as a promising candidate for intervention in hypertensive cardiac remodeling. Inhibition of CCL5 signaling attenuates structural and functional abnormalities of the heart, including fibrosis, hypertrophy, and impaired function, triggered by Ang II. This research also represents the first exploration of CCL5's role in regulating platelet inflammatory activation and further investigates the CCL5-dependent platelet-primed inflammatory cascade, including promoting inflammatory cell recruitment and M2 macrophage polarization. Mechanistically, CCL5 promotes platelet-derived TGF-β1 secretion through the NF-κB signaling pathway *via* CCR1 and CCR3 receptors, thereby exerting pro-inflammatory and pro-fibrotic effects. Taken together, our results delineate the multiple functions of CCL5 in modulating platelet activation and macrophage polarization. These insights indicate that therapeutic intervention against this pathway and its associated platelet-macrophage crosstalk could represent a novel approach to mitigate inflammatory responses and ameliorate cardiac remodeling.

## Supplementary Material

Supplementary figures and table.

## Abbreviations

Ang II: angiotensin II
CCL5 KO: CCL5 knockout
WT: wild-type
rmCCL5: recombinant CCL5
TAC: transverse aortic constriction
CCL5: C-C chemokine motif ligand 5
NF-κB: nuclear factor-kappa B
TGF-β1: transforming growth factor-beta1
GSEA: gene set enrichment analysis
KEGG: kyoto encyclopedia of genes and genomes
GO: gene ontology
GSVA: gene set variation analysis
ASA: aspirin
CPG: clopidogrel
PRP: platelet-rich plasma
EF: ejection fraction
FS: fractional shortening
ESAWT: end-systolic anterior wall thickness
ESPWT: end-systolic posterior wall thickness
PCA: principal component analysis
DEGs: differentially expressed genes
THR: thrombin
