# Supplementary material for: CCL5 promotes angiotensin II-induced cardiac remodeling through regulation of platelet-driven M2 macrophage polarization
Source: Theranostics. 2026 Jan 1;16(2):689–711. doi: 10.7150/thno.112163 (PMC12674933; doi:10.7150/thno.112163)
Supplement: Supplementary file 1 — Supplementary figures and table. [file thnov16p0689s1.pdf]

CCL5 promotes angiotensin II-induced cardiac remodeling through regulation of platelet-driven M2 macrophage polarization

Authors:

Silin Lv[1, 2], Mingxuan Zhou[1, 2], Tiegang Li[1, 2], Zifan Zeng[1, 2], Zheng Yan[1, 2], Yufang Hou[1, 2], Liu Yang[1, 2], Fang Zhang[1, 2], Wenyi Zhao[1, 2], Yixin Zhou[1, 2], Min Yang[1, 2]*

Affiliations: [1] State Key Laboratory of Bioactive Substance and Function of Natural Medicines, Institute of Materia Medica, Chinese Academy of Medical Sciences and Peking Union Medical College, Beijing 100050, China.

[2] State Key Laboratory of Digestive Health, Institute of Materia Medica, Chinese Academy of Medical Sciences and Peking Union Medical College, Beijing 100050, China.

Correspondence to: Min Yang*, State Key Laboratory of Bioactive Substance and Function of Natural Medicines, State Key Laboratory of Digestive Health, Institute of Materia Medica, Chinese Academy of Medical Sciences and Peking Union Medical College, No. 2 Nanwei Road, Beijing 100050, China.

E-mail: minyang@imm.ac.cn

Tel: +86(10)63015871

Fax: +86(10)63017757

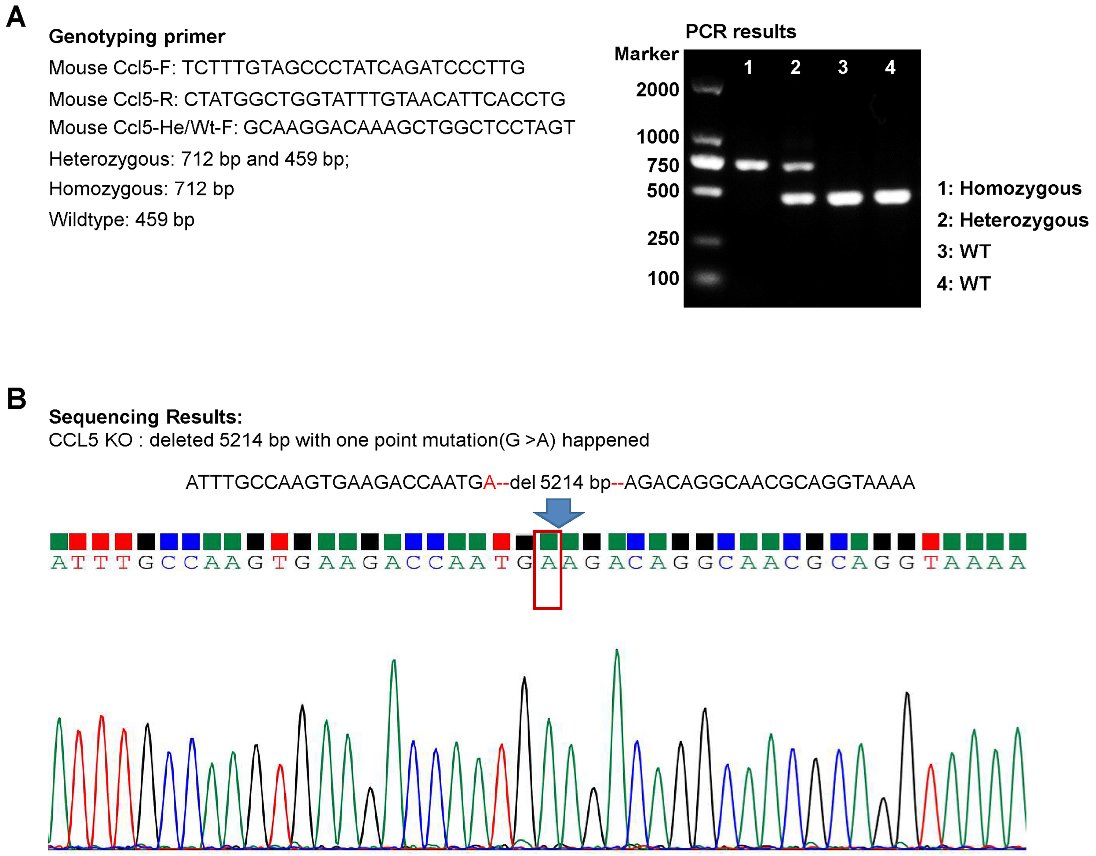

Figure S1 Genotypes and DNA sequencing of CCL5 KO mice. A) PCR genotyping results: 1, Homozygous; 2, Heterozygous; 3, WT; 4, WT. **B)** Sequencing results: A 5214 bp deletion with a point mutation (G>A) was introduced between exons 1 and 3 of the CCL5 genomic DNA, leading to early termination of translation.

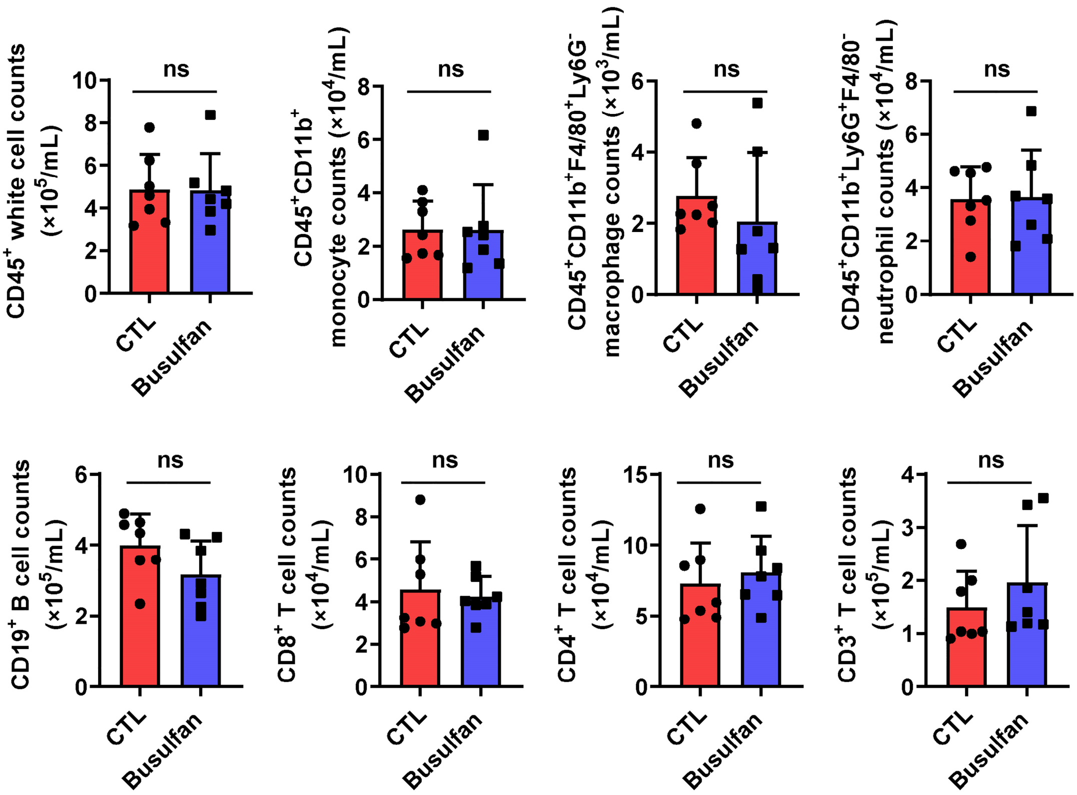

Figure S2 The administration of busulfan does not alter the number of peripheral blood leukocytes. Mice were administrated with busulfan (20 mg/kg) or vehicle. Flow cytometric analysis was used for peripheral blood leukocyte counts (n = 7). NS indicating no significance.

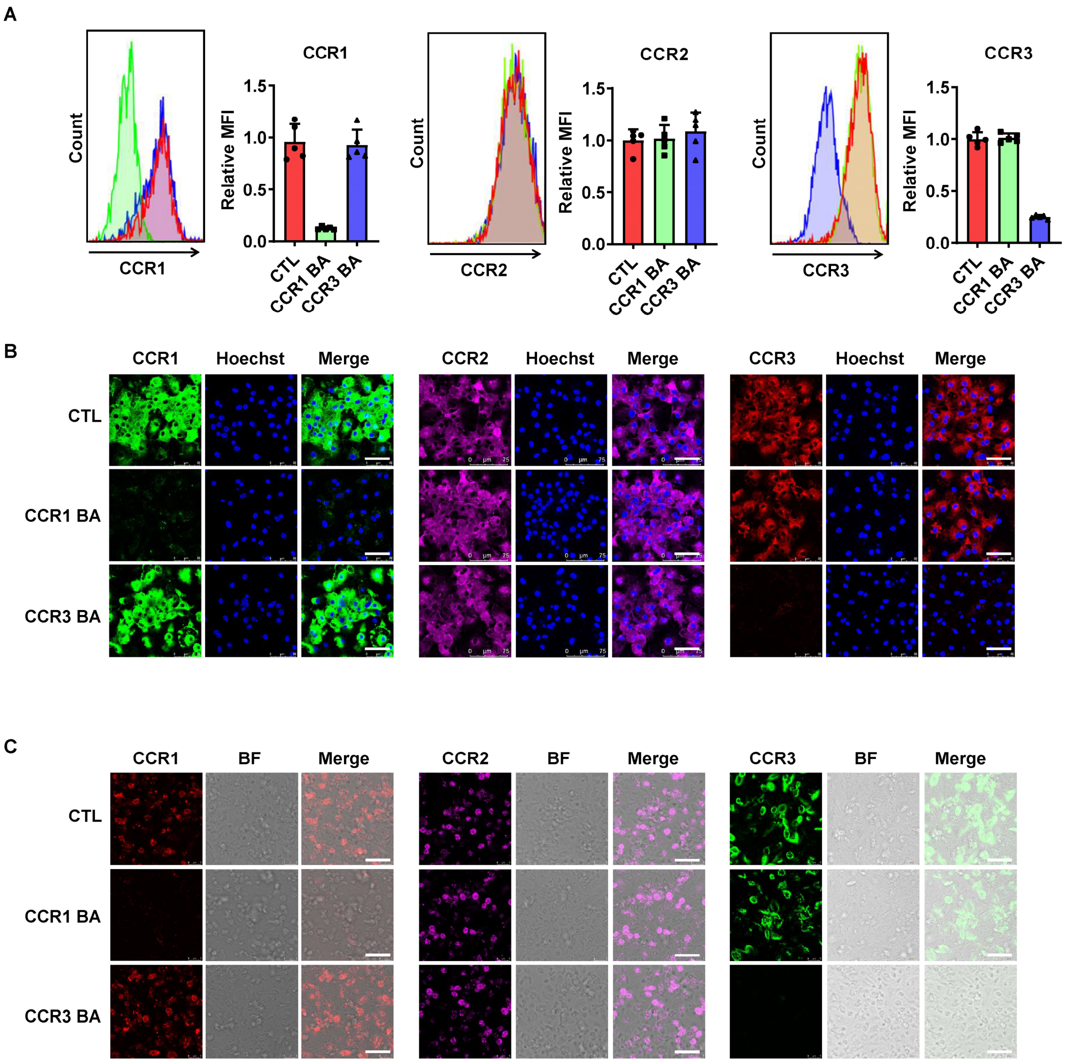

Figure S3 The specificity of CCR1 and CCR3 blocking antibodies. The detection of CCR1, CCR2, and CCR3 on bone marrow-derived macrophages (BMDMs) following treated with CCR1 blocking antibody (CCR1 BA) and CCR3 blocking antibody (CCR3 BA) was performed using flow cytometry (**A**) and immunofluorescence staining (**B**) (n = 5). The presence of CCR1, CCR2, and CCR3 on platelets after incubation with CCR1 and CCR3 blocking antibodies was assessed *via* immunofluorescence staining (**C**) (n = 5). CCR1 blocking antibody successfully blocked CCR1 on both BMDMs and platelets, and showed no effects on CCR2 and CCR3. CCR3 blocking antibody only blocked CCR3, but had no significant effects on CCR1 and CCR2 on both BMDMs and platelets. Data are presented as mean ± standard deviation, with n representing the number of animals. * $P < 0.05$, ** $P < 0.01$, *** $P < 0.001$.

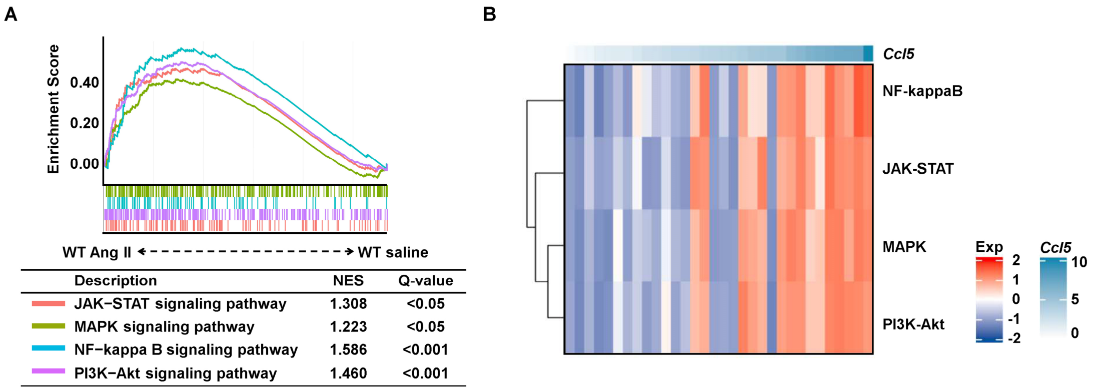

Figure S4 Signaling pathways regulated CCL5 expression were activated in Ang II-infused hearts. A) GSEA analysis of signaling pathways regulating CCL5 expression was performed on GEO datasets, comparing WT hearts following a 7-day infusion of either Ang II or vehicle (n = 32). **B)** Correlation between *Ccl5* gene expression and GSVA scores for NF-kappa B, JAK-STAT, MAPK and PI3K-Akt signaling pathways in hearts following a 7-day infusion of either Ang II or vehicle, using GEO datasets (n = 32).

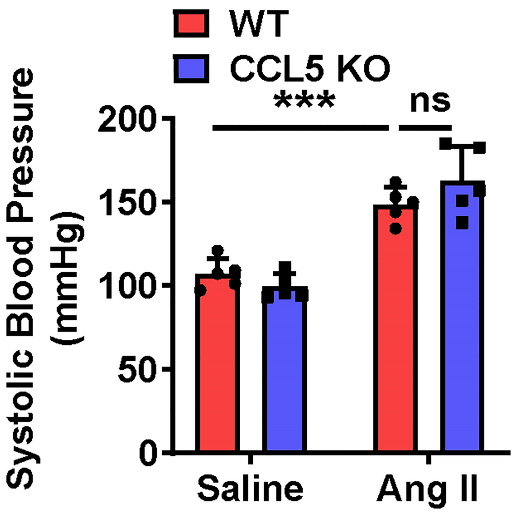

Figure S5 CCL5 KO does not affect Ang II-evaluated systolic blood pressure. Blood pressure measured *via* tail-cuff system (n = 5).

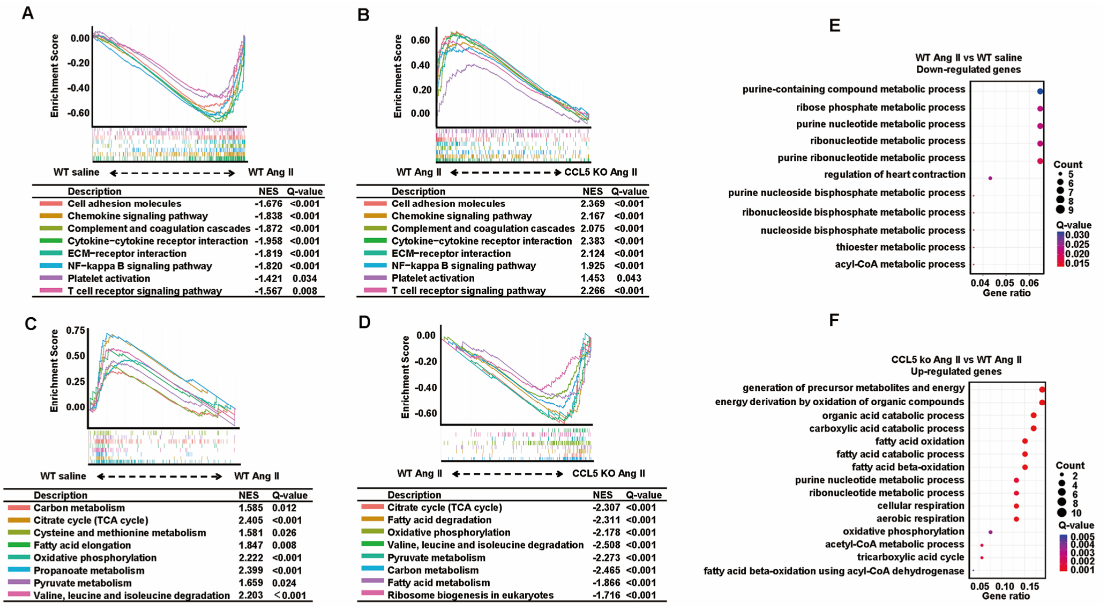

Figure S6 Signaling pathways dysregulated by CCL5 during hypertensive cardiac remodeling. WT and CCL5 KO mice were subjected to a 7-day infusion of saline or Ang II. Hearts were collected for RNA-seq analysis (n = 3 per group). **A-D)** Significantly enriched pathways identified through gene set enrichment analysis (GSEA). **E)** Gene Ontology (GO) analysis of biological processes for genes downregulated in WT Ang II hearts compared to WT saline hearts. **F)** GO analysis of biological processes for genes upregulated in CCL5 KO Ang II hearts compared to WT Ang II hearts. A Q value < 0.05 was considered statistically significant.

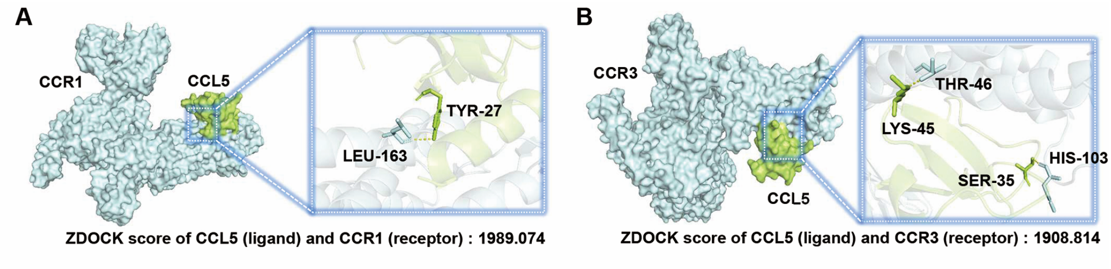

Figure S7 Molecular docking analysis the interaction of CCL5 with CCR1 and CCR3. A) CCL5 (ligand) and CCR1 (receptor) formed hydrogen bonds through amino acid residue sites of TYR-27 and LEU-163, with ZDOCK score of 1989.074. **B)** CCL5 (ligand) and CCR3 (receptor) formed hydrogen bonds through amino acid residue sites of LYS-45 and THR-46, SER-35 and HIS-103, with ZDOCK score of 1908.814.

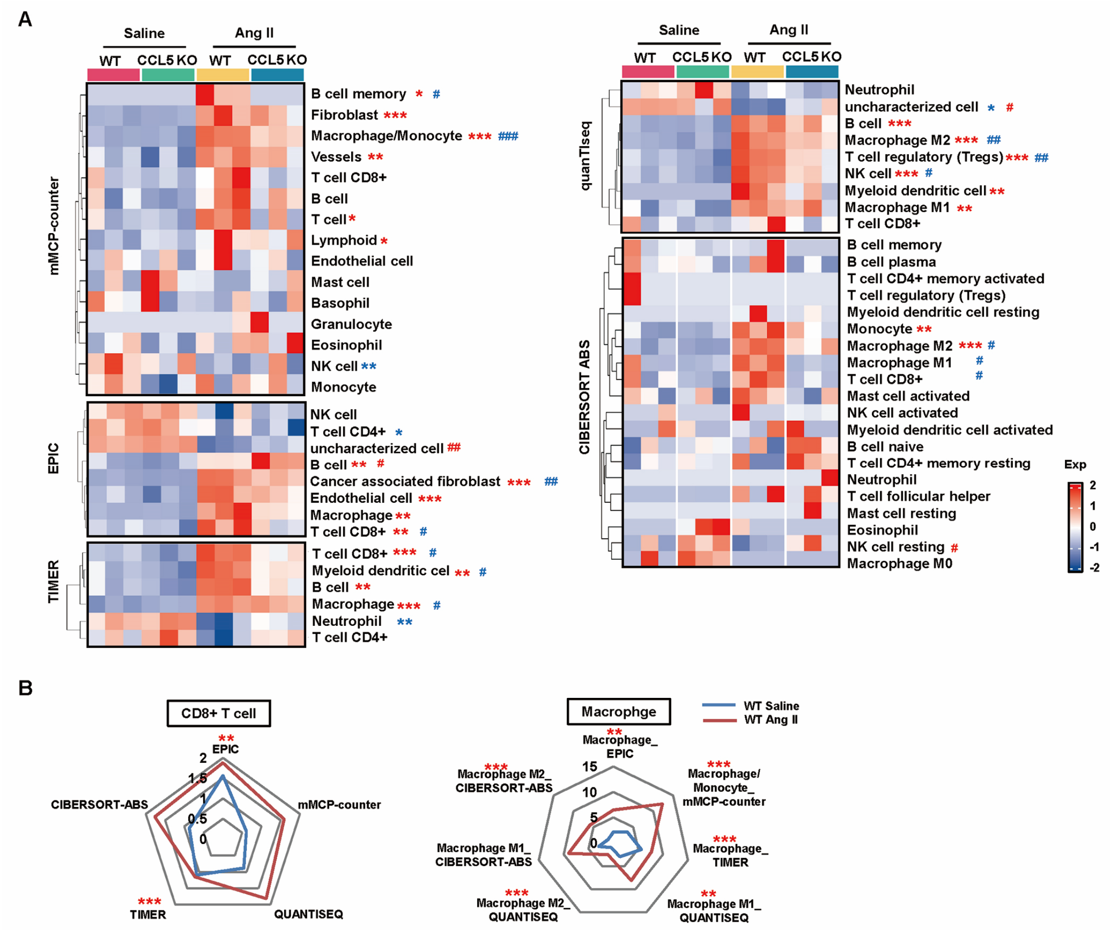

Figure S8 Immune infiltration regulated by CCL5 in the heart. WT and CCL5 KO mice were subjected to a 7-day infusion of saline or Ang II. Hearts were collected for RNA-seq analysis (n = 3 for each group). **A)** Immune infiltration of immune cells obtained by mMCP-counter, EPIC, TIMER, QUANTISEQ, and CIBERSORT ABS. **B)** Radar plot demonstrating the extent of CD8+ T cells and macrophages, including M1 and M2, comparing WT Ang II hearts to WT saline hearts using five algorithms. *P adj < 0.05, ** P adj < 0.01, ***P adj < 0.001 represents the WT Ang II group compared to the WT saline group; # P adj < 0.05, ## P adj < 0.01, ### P adj < 0.001 represents the CCL5 KO Ang II group compared to the WT Ang II group. Upregulated in red, and downregulated in blue.

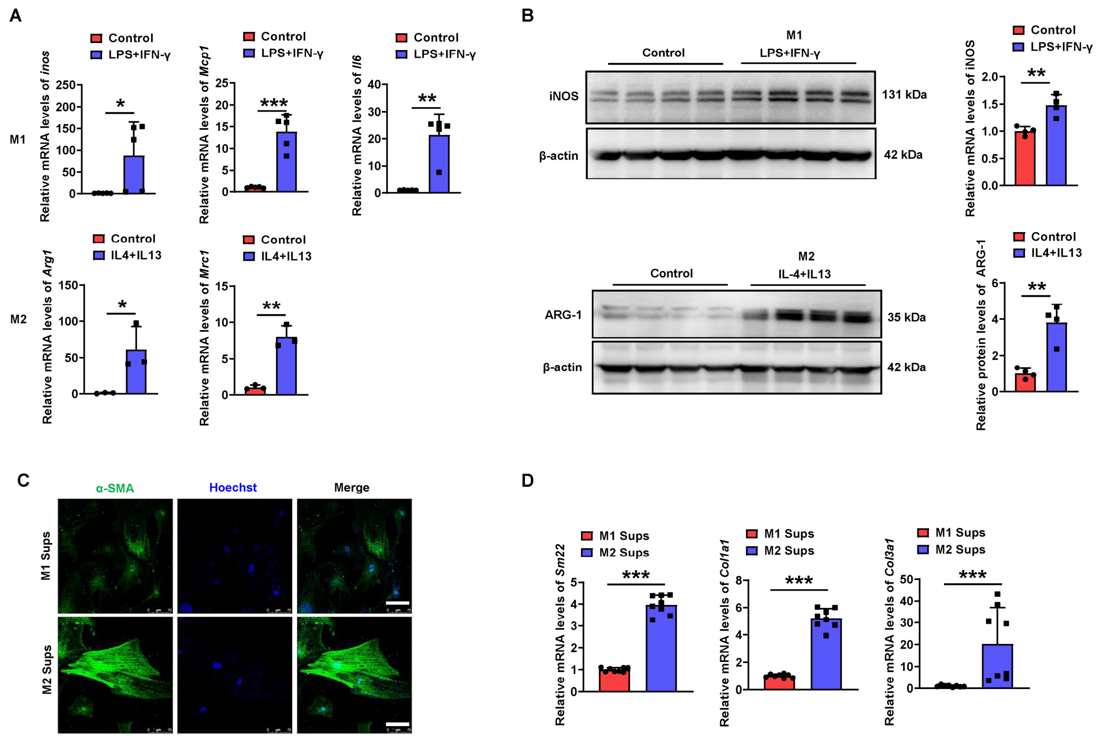

Figure S9 M2 macrophages have a stronger ability to promote myofibroblast differentiation compared to M1 macrophages. RAW264.7 cells were classically activated (M1 condition) by LPS (100 ng/ml) and IFN-gamma (20 ng/mL), and alternatively activated (M2 condition) with IL-4 (20 ng/mL) and IL-13 (20 ng/mL) or received media alone (M0 condition) *in vitro* for 48 hours, and proliferated in new media without cytokines for another 24 hours. The supernatants were collected for fibroblast stimulation. Generation of M1 and M2 macrophages were verified by RT-PCR (**A**) (n = 3) and western blotting (**B**) (n = 4). Myofibroblast differentiation were detected by immunofluorescence staining of α-SMA (Scale bar: 75 μm) (**C**) (n = 5) and RT-PCR of *Sm22*, *Col1a1* and *Col3a1* (**D**) (n = 8). Data are presented as mean ± standard deviation, with * indicating P < 0.05, ** P < 0.01, *** P < 0.001.

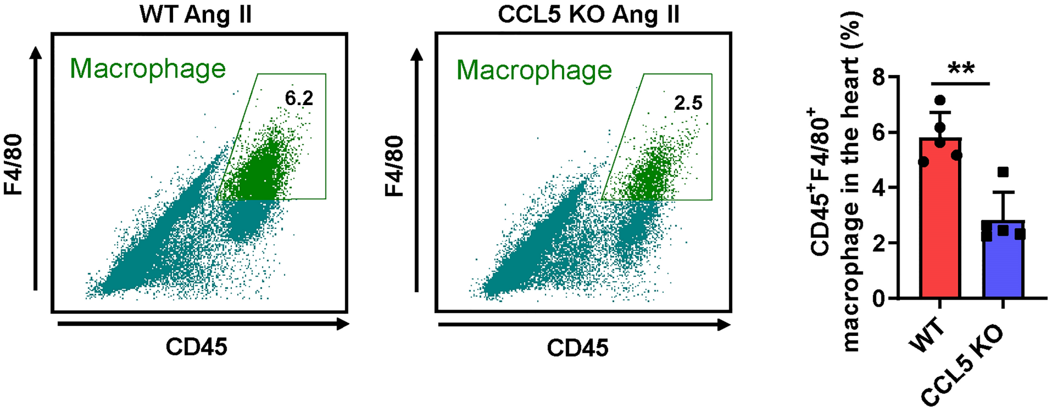

Figure S10 CCL5 deficiency attenuates Ang II-induced cardiac macrophage accumulation. WT and CCL5 KO mice were subjected to a 7-day infusion of saline or Ang II. Flow cytometric analysis was used to calculate the proportion of macrophage ($CD45^{+}F4/80^{+}$) among total cells in the heart (n = 5). Data are presented as mean ± standard deviation, with n representing the number of animals. * P < 0.05, ** P < 0.01, *** P < 0.001.

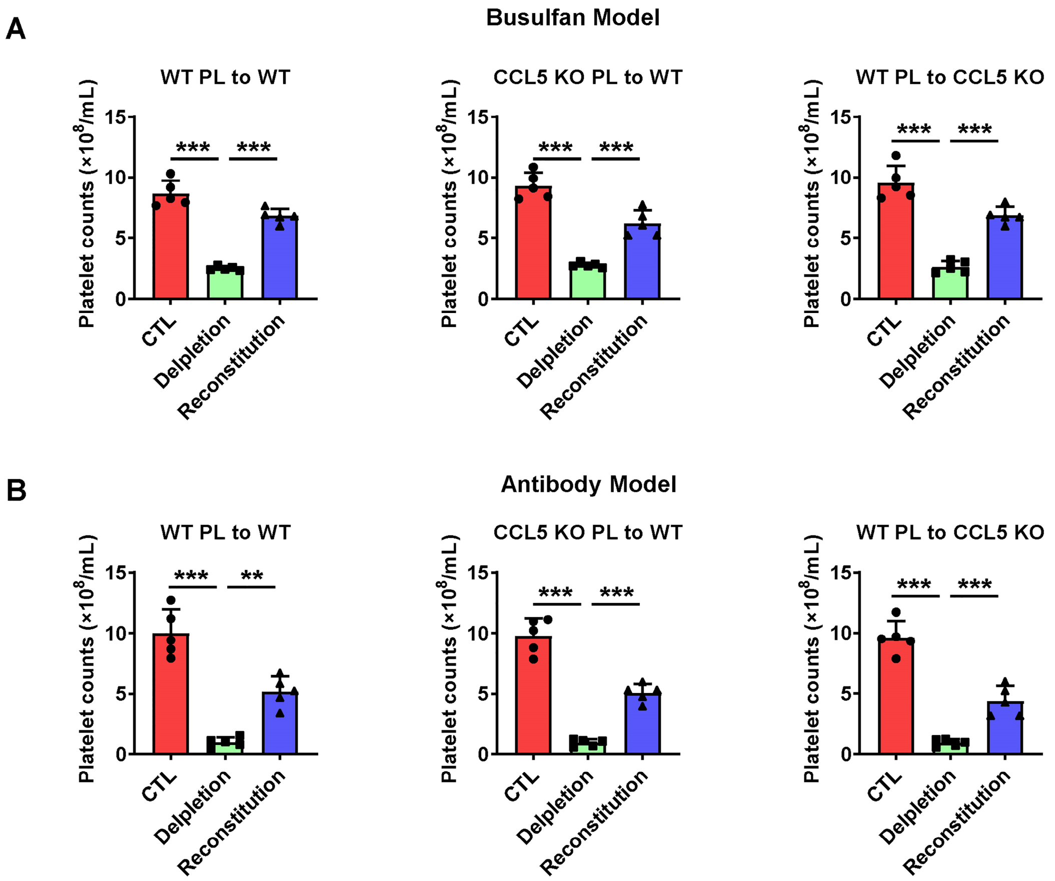

Figure S11 Platelet counts of mice in the two distinct platelet depletion/reconstitution model. Platelet counts before depletion, after depletion, and after reconstitution in the busulfan model (**A**) and antibody model (**B**) were assessed to confirm the successful depletion and reconstitution of platelets (n = 5). Data are presented as mean ± standard deviation, with * indicating $P < 0.05$, ** $P < 0.01$, *** $P < 0.001$.

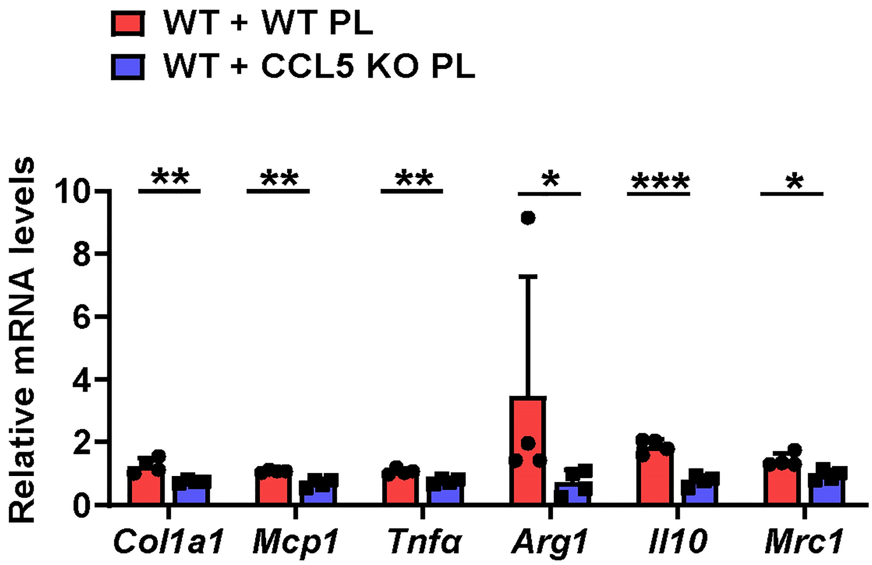

Figure S12 CCL5-deficient platelets attenuate Ang II-induced cardiac fibrosis, inflammation, and M2 macrophage gene expression. Recipient WT mice were platelet depleted by chemotherapy drug busulfan, then reconstituted platelets from WT or CCL5 KO mice prior to Ang II infusion. Hearts were collected for molecular analysis. Gene expression of fibrosis markers (*Col1a1*), inflammatory markers (*Mcp1*, *Tnfα*), and M2 macrophage markers (*Arg1*, *Il10*, *Mrc1*) were assessed by RT-PCR (n = 4). Data are presented as mean ± standard deviation, with * indicating $P < 0.05$, ** $P < 0.01$, *** $P < 0.001$.

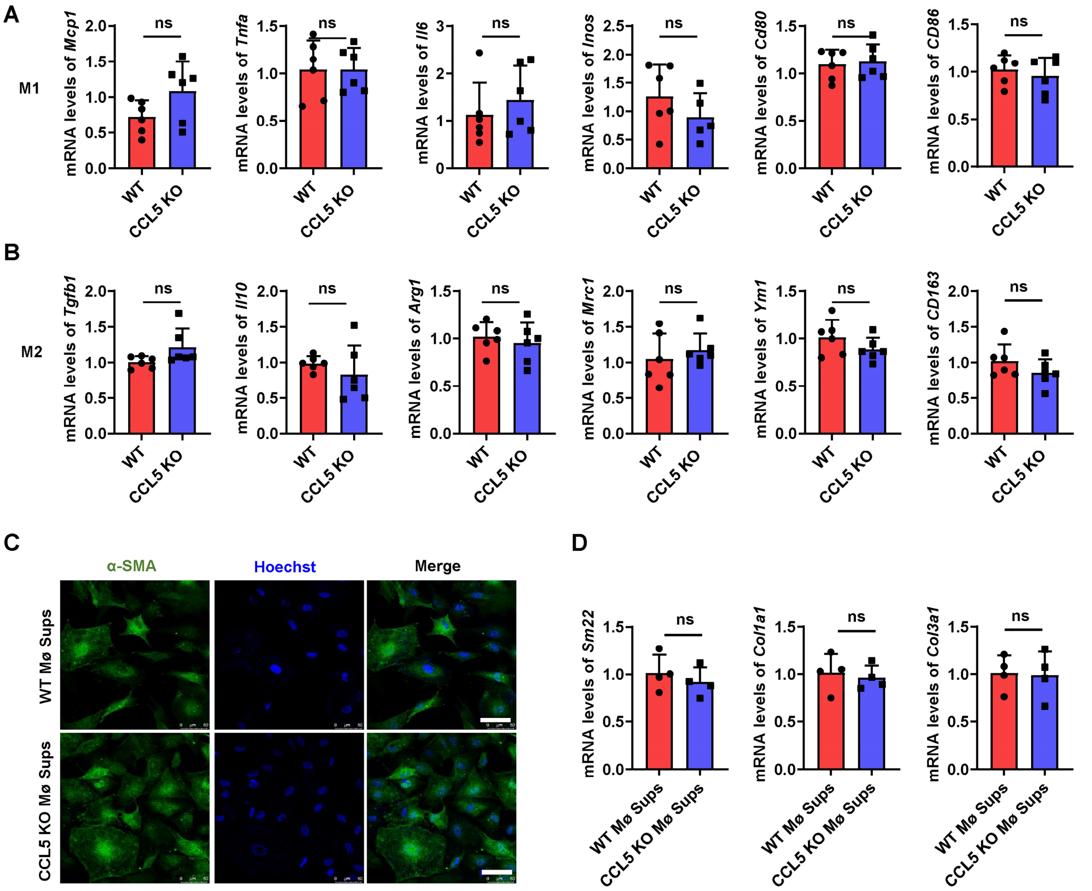

Figure S13 Macrophage-intrinsic CCL5 deficiency has no effects on macrophage polarization. BMDM from WT and CCL5 KO mice were stimulated with Ang II (100 nM) for 48 hours *in vitro*, and the supernatant were collected for fibroblast stimulation. Genes expression of M1 (*Mcp1*, *Tnfa*, *Il6*, *Inos*, *Cd80* and *Cd86*) (**A**) and M2 (Tgfb1, *Il10*, *Arg1*, *Mrc1*, *Ym1*) (**B**) macrophages were detected by RT-PCR (n = 6). Myofibroblast differentiation were detected by immunofluorescence of α-SMA (Scale bar: 50 μm) (**C**) (n = 5) and RT-PCR of *Sm22*, *Col1a1* and *Col3a1*) (**D**) (n = 4). Data are presented as mean ± standard deviation, with * indicating P < 0.05, ** P < 0.01, *** P < 0.001. NS indicating no significance.

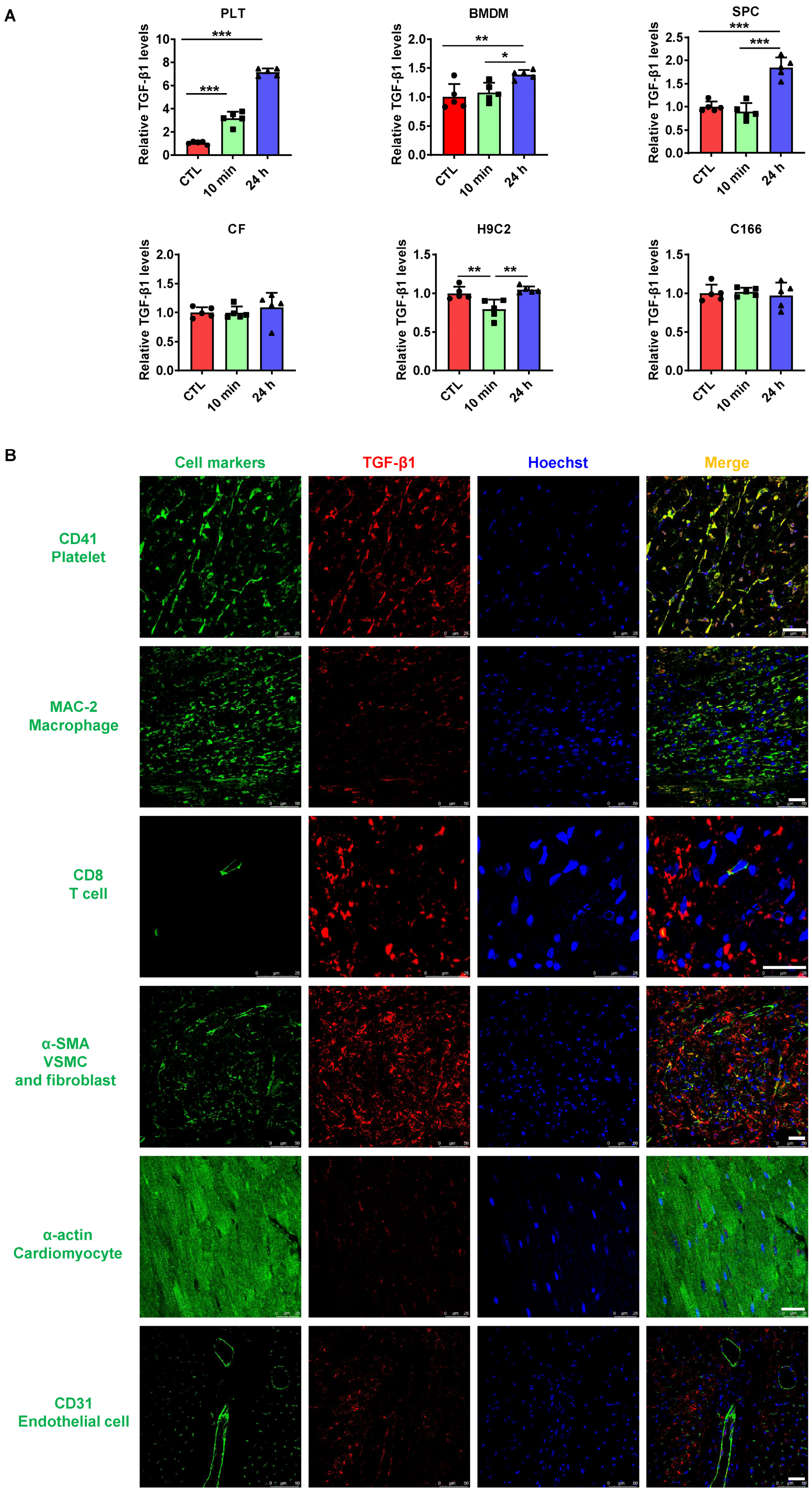
A
PLT
BMDM
SPC
CF
H9C2
C166
Relative TGF-β1 levels
CTL
10 min
24 h
B
Cell markers
TGF-β1
Hoechst
Merge
CD41
Platelet
MAC-2
Macrophage
CD8
T cell
α-SMA
VSMC
and fibroblast
α-actin
Cardiomyocyte
CD31
Endothelial cell

Figure S14 Cardiac TGF-β1 in the early phase of Ang II infusion is primarily derived from platelets. A) Washed platelets (PLT), bone marrow-derived macrophages (BMDMs), splenocytes (SPC), cardiac fibroblasts (CF), H9C2 (cardiomyocytes), and C166 (endothelial cells) were stimulated with ADP (10 μM) and Ang II (100 nM) for 10 minutes and 24 hours. TGF-β1 levels in the supernatants were measured by ELISA (n = 5). **B)** WT mice were subjected with a 3-day Ang II. Immunofluorescence showed the distribution of platelets (CD41, green), macrophages (MAC-2, green), T cells (CD8, green), vascular smooth muscle cells (α-SMA, green), fibroblasts (α-SMA, green), cardiomyocytes (α-actin, green), endothelial cells (CD31, green), and TGF-β1 (red) in the heart (n = 5; scale bar: 25 μm). Data are presented as mean ± standard deviation, with * indicating $P < 0.05$, ** $P < 0.01$, *** $P < 0.001$.

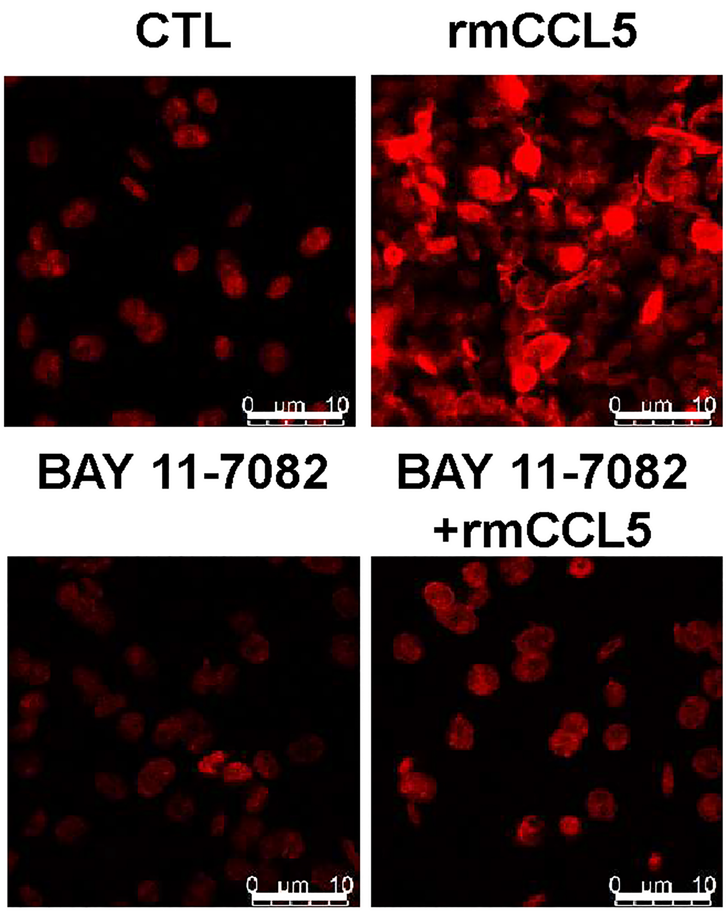

Figure S15 CCL5 promotes platelet F-actin polymerization *via* NF-κB signaling pathway. Before being stimulated with rmCCL5 (100 ng/mL) and ADP (5 μM) for 10 minutes, WT platelets were preincubated for 15 minutes in the presence or absence of BAY 11-7082 (1 μM), an inhibitor of NF-κB. F-actin polymerization was detected by phalloidin-FITC (n = 5; Scale bar: 10 μm).

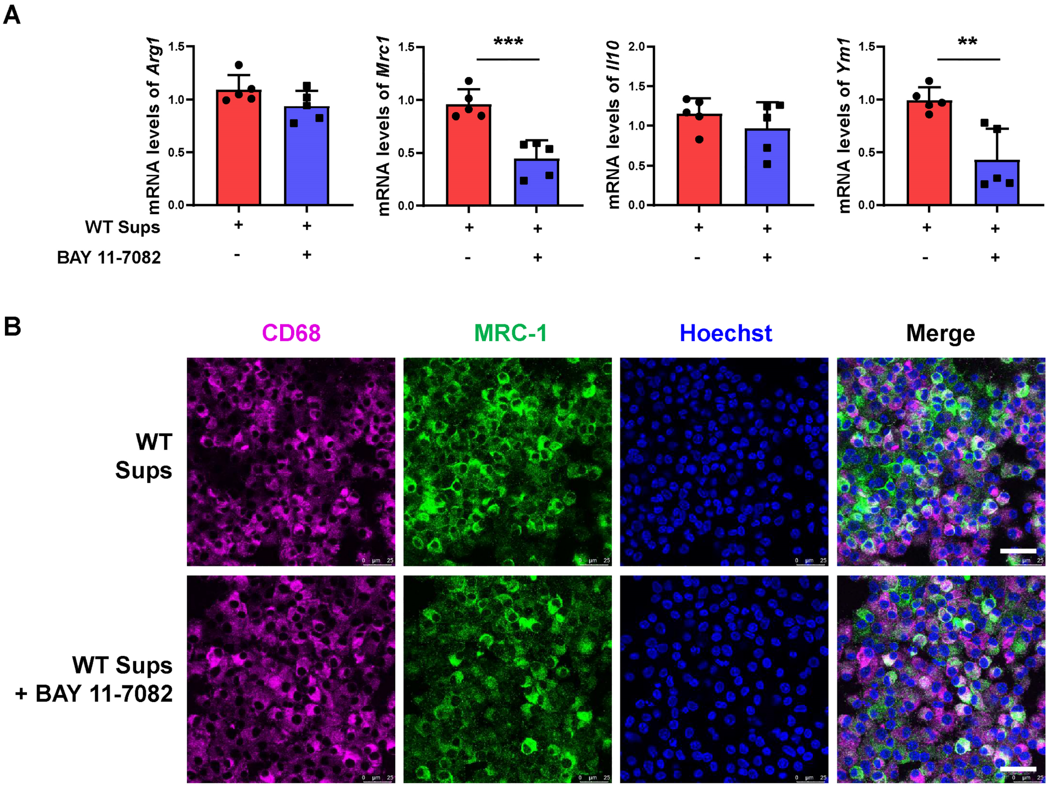

Figure S16 NF-κB inhibition attenuates the induction of M2 polarization by WT platelet supernatant. BMDMs were treated with the NF-κB inhibitor BAY 11-7082 (10 nM) or vehicle control prior to a 48-hour co-culture with supernatants derived from ADP and Ang II-stimulated WT platelets. **A)** Expression of M2 marker genes (*Arg1*, *Il10*, *Mrc1*, *Ym1*) in macrophages, as determined by RT-qPCR (n = 5). **B)** Protein level of MRC1 was evaluated by immunofluorescence staining (n = 5; scale bar: 25 μm). Data are presented as mean ± standard deviation, with * indicating P < 0.05, ** P < 0.01, *** P < 0.001.

Supporting Information Table 1 Primer sequences for RT-PCR.

Gene name	Forward primer	Reverse primer
Mus:		
Ccl5	CTTCCCTGTCATTGCTTGCTC	CCGAGTGGGAGTAGGGGATT
Col1a1	GAGTACTGGATCGACCCTAACCA	GACGGCTGAGTAGGGAACACA
Col3a1	TCCCCTGGAATCTGTGAATC	TGAGTCGAATTGGGGAGAAT
Anf	CACAGATCTGATGGATTTCAAGA	CCTCATCTTCTACCGGCATC
Bnp	GAAGGTGCTGTCCCAGATGA	CCAGCAGCTGCATCTTGAAT
Mcp1	CCTGGATCGGAACCAAATGA	ACCTTAGGGCAGATGCAGTTTTA
Tnfα	AAGGGGATTATGGCTCAGGGT	TTGATGGTGGTGCATGAGAGG
Il6	GCTACCAAACTGGATATAATCAGGA	CCAGGTAGCTATGGTACTCCAGAA
Inos	ACATCGACCCGTCCACAGTAT	CAGAGGGGTAGGCTTGTCTC
Cd80	ACCCCCAACATAACTGAGTCT	TTCCAACCAAGAGAAGCGAGG
Cd86	CTGGACTCTACGACTTCACAATG	AGTTGGCGATCACTGACAGTT
Arg1	ACACTGACATCAACACTCCCC	AGGAGAAAGGACACAGGTTGC
Il10	CTTGGGAAGAGAAACCAGGGAG	TGGGGGATGACAGTAGGGGA
Tgfb1	CTGCTGACCCCCACTGATAC	AGCCCTGTATTCCGTCTCCT
Mrc1	GCTACACTGGTGGTAGGTCTC	CTGAGTGGCTTACGTGGTTG
Ym1	CAGGTCTGGCAATTCTTCTGAA	GTCTTGCTCATGTGTGTAAGTGA
Cd163	ATGGGTGGACACAGAATGGTT	CAGGAGCGTTAGTGACAGCAG
Ccr1	GGCAAATGGCTCACTGGAAA	TCTTTCCTTTCATCATGTGAGTTCC
Ccr3	TGAGCAGAACAATGAAGAGGT	ACAAGATGAGGGGACTATGACC
Gapdh	TGGAGAAACCTGCCAAGTATGA	GGTCCTCAGTGTAGCCCAAG
Rat:		
Col1a1	GGAGAGAGCATGACCGATGG	GCTACGCTGTTCTTGCAGTG
Col3a1	GGACCAGGCAATGATGGGAA	CAGGGAAACCCATGACACCA
α-Sma	TTGTCCACCGCAAATGCTT	TGAAGGCGCTGATCCACAAA
Sm22	CGATGGACACTACCGTGGAG	TTTGAAGGCCAATGACGTGC
Gapdh	ATTCCATCCCAGACCCCATAAC	GCAGCGAACTTTATTGATGGTAT
